# FANCM Limits Meiotic Crossovers in Brassica Crops

**DOI:** 10.3389/fpls.2018.00368

**Published:** 2018-03-23

**Authors:** Aurélien Blary, Adrián Gonzalo, Frédérique Eber, Aurélie Bérard, Hélène Bergès, Nadia Bessoltane, Delphine Charif, Catherine Charpentier, Laurence Cromer, Joelle Fourment, Camille Genevriez, Marie-Christine Le Paslier, Maryse Lodé, Marie-Odile Lucas, Nathalie Nesi, Andrew Lloyd, Anne-Marie Chèvre, Eric Jenczewski

**Affiliations:** ^1^Institut Jean-Pierre Bourgin, Institut National de la Recherche Agronomique, AgroParisTech, Centre National De La Recherche Scientifique, Université Paris-Saclay, Versailles, France; ^2^IGEPP, Institut National de la Recherche Agronomique, Agrocampus Ouest, Université de Rennes 1, Le Rheu, France; ^3^EPGV US 1279, Institut National de la Recherche Agronomique, CEA-IG-CNG, Université Paris-Saclay, Evry, France; ^4^Institut National de la Recherche Agronomique UPR 1258, Centre National des Ressources Génomiques Végétales, Castanet-Tolosan, France

**Keywords:** FANCM, Translational biology, *Brassica*, meiotic crossover, TILLING, plant breeding, polyploidy

## Abstract

Meiotic crossovers (COs) are essential for proper chromosome segregation and the reshuffling of alleles during meiosis. In WT plants, the number of COs is usually small, which limits the genetic variation that can be captured by plant breeding programs. Part of this limitation is imposed by proteins like FANCM, the inactivation of which results in a 3-fold increase in COs in *Arabidopsis thaliana*. Whether the same holds true in crops needed to be established. In this study, we identified EMS induced mutations in FANCM in two species of economic relevance within the genus *Brassica*. We showed that CO frequencies were increased in *fancm* mutants in both diploid and tetraploid *Brassicas, Brassica rapa* and *Brassica napus* respectively. In *B. rapa*, we observed a 3-fold increase in the number of COs, equal to the increase observed previously in *Arabidopsis*. In *B. napus* we observed a lesser but consistent increase (1.3-fold) in both euploid (AACC) and allohaploid (AC) plants. Complementation tests in *A. thaliana* suggest that the smaller increase in crossover frequency observed in *B. napus* reflects residual activity of the mutant C copy of FANCM. Altogether our results indicate that the anti-CO activity of FANCM is conserved across the *Brassica*, opening new avenues to make a wider range of genetic diversity accessible to crop improvement.

## Introduction

Meiotic recombination is essential for proper chromosome segregation and reshuffling of genetic information through the formation of Cross-Overs (COs); i.e., reciprocal exchanges of genetic material between homologous chromosomes. Meiotic recombination plays both a direct and an indirect role in plant genome evolution because of its inherent mutagenic nature (Rattray et al., 2015) and its influence on selection (Tiley and Burleigh, 2015). It is also central to plant breeding (Wijnker and de Jong, 2008) as it produces new combinations of alleles on which selection can act. Accordingly, an increase in CO frequencies is predicted to result in a better response to selection (McClosky and Tanksley, 2013). Yet the number of COs is low in most species, rarely exceeding 2–3 per chromosome (Mercier et al., 2015).

Meiotic recombination is initiated by programmed double strand breaks (DSBs) (Keeney et al., 1997) that can be repaired as COs through two pathways. The first pathway, which forms the majority of COs (i.e., “class I” COs), is dependent on a group of proteins initially identified in *S. cerevisiae* and collectively called ZMMs (Zip1-4, Msh4/Msh5, and Mer3). In *A. thaliana, zmm* mutants, including *Atmsh4* and *Atmsh5*, show severely reduced fertility due to a decrease in CO frequency (~15% of the WT CO level) (Higgins et al., 2004, 2008). The distribution of class I COs ensures one obligate CO per pair of homologous chromosomes and is subject to interference; this means that the presence of one CO reduces the probability of observing another CO in the vicinity. The second pathway, which is secondary in WT meiosis, depends, at least in part, on the endonuclease MUS81; the resulting class II COs are randomly distributed (i.e., not affected by CO interference) and far more difficult to mark cytologically (Anderson et al., 2014). The vast majority of DSBs however, are repaired as non-reciprocal exchanges of genetic material, termed non Cross-Overs (NCOs). Because the number of DSBs vastly outnumbers COs, negative regulators of CO frequency have been hypothesized. In *Arabidopsis thaliana*, genetic screens designed to identify these negative regulators have been carried out and have identified genes in three distinct pathways that limit class II COs (Crismani et al., 2012; Girard et al., 2014, 2015; Séguéla-Arnaud et al., 2015; Fernandes et al., in review).

The first anti-CO protein identified through these screens was FANCM (Fanconi Anemia Complementation Group M) (Crismani et al., 2012). FANCM has long been recognized as a core component of the Fanconi Anemia pathway, a network of at least 22 proteins identified in human that preserve genome stability by promoting the processing of interstrand crosslinks (Wang and Smogorzewska, 2015). In addition to a C-terminal ERCC4-like nuclease domain and a tandem helix–hairpin–helix (HhH)_2_ domain, FANCM consists of an N-terminal bipartite SF2 helicase domain (composed of a DEXDc and a HELICc domain) (Whitby, 2010). *FANCM* orthologs have now been identified in various eukaryotes in which they do not always play the exact same role (Lorenz et al., 2012).

Studies in *A. thaliana* showed that AtFANCM regulates somatic and meiotic recombination (Knoll and Puchta, 2011; Crismani et al., 2012). During meiotic recombination, FANCM is thought to promote NCO formation through the SDSA pathway (Crismani et al., 2012). FANCM acts as a landing pad for multiple Fanconi Anemia associated proteins (Vinciguerra and D'Andrea, 2009). In Arabidopsis, only FANCM direct DNA-binding cofactors MHF1 and MHF2 were shown to contribute to the FANCM anti-CO activity (Girard et al., 2014). The SF2 helicase domain of AtFANCM appears to be critical for its anti-CO activity. Mutations in well-conserved residues of the DEXDc and a HELICc domains were indeed shown to increase MUS81-dependent CO formation in *fancm* single mutants (Crismani et al., 2012). This increase is so huge that it restores bivalent formation in *zmm* CO-defective mutants to a level indistinguishable from WT.

The boost in COs observed in *atfancm* mutants, which can be up to 3.6-fold in some intervals, could be of great interest for plant breeding. Yet, to the best of our knowledge, the effect of FANCM on CO formation has never been assessed in a crop species. The present study aimed to fill this gap using *Brassica* crops as models.

In addition to the model species *A. thaliana*, the *Brassicaceae* family includes many diploid and polyploid crops (e.g., *B. rapa, B. oleracea, B. napus, B. juncea*) that show a rich diversity of morphotypes (Cheng et al., 2014). Although many of these species can be used as vegetable, fodder, oilseed or even as ornamental crops, diploid *B. rapa* (chinese cabbage, turnip, pak choi…) and *B. oleracea* (cabbage, Brussels sprouts, broccoli, cauliflower…) are often referred to as leaf vegetables while allotetraploid *B. napus* (oilseed rape or canola) is mainly cultivated as an oilseed crop. *B. napus* (AACC; 2*n* = 38) arose from multiple hybridization events between the ancestors of modern *B. oleracea* (CC; 2*n* = 18) and *B. rapa* (AA; 2*n* = 20). Because the progenitors of *B. napus* have experienced a whole-genome triplication (WGT) before hybridization (Lysak et al., 2005), every gene in *A. thaliana* could possibly have up to 6 homologs in *B. napus*. Such a high number of homologs is rarely observed however, as fractionation, the process by which duplicate genes are lost (Freeling, 2009; Woodhouse et al., 2010), starts right after the onset of WGD (Li Z. et al., 2016). The trend is especially strong for meiotic recombination genes that return to a single copy more rapidly than genome-wide average in angiosperms (Lloyd et al., 2014).

Intense selection in *Brassica* resulted in a notable decline in genetic diversity in modern cultivars of *B. napus, B. rapa*, and *B. oleracea* (Hasan et al., 2006; Qian et al., 2014; Cheng et al., 2016). Increasing meiotic crossovers in *Brassica* crops could thus be of great interest to generate novel genetic combinations and expand the range of genotypes available in these cultivated species. In this study, we explore the anti-CO activity of FANCM in two *Brassica* species, diploid *B. rapa*, and allotetraploid *B. napus*, as a proof-of-concept for all other crops in this family.

## Materials and methods

### Development of a mutagenized population for *Brassica napus*

Seeds from *Brassica napus* L. cv. *Tanto* (double-low spring cultivar, INRA Rennes, France) were immersed into a 0.5% EMS solution overnight under moderate shaking (200 rpm). The treated seeds were rinsed three times for 5 min in a solution of 1 M sodium thiosulfate, twice for 5 min in distilled water and then briefly dried onto a paper towel before being disposed on a water-imbibed Whatman filter paper in Petri dishes. Seeds were allowed to germinate at room temperature for two days in the dark and to elongate for two additional days under 16 h light/8 h dark.

Seedlings from treated seeds (hereafter called the M1 generation) were transferred into individual pots filled with a mixture of 20% black peat, 70% white peat and 10% perlit as substrate (Haasnoot Substraten, Zaltbommel, NL) and grown in the glasshouse (16 h light at 22°C/8 h dark at 18°C; 200 μmol.m^−2^.s^−1^ light intensity at the plant level). Six-leaf plants were vernalized for four weeks to ensure correct and homogenous flowering and vernalized plants were transferred into a tunnel. At flowering, inflorescences on the primary raceme were covered with a selfing bag to avoid cross pollination while the branches were regularly cut. Pods from the main inflorescences were harvested at ~1,000 growing degree (°C) days after flowering and the collected seeds constituted the M2 seed lots, each arising from a single mutagenized M1 plant. The whole mutagenized population (hereafter called the RAPTILL population) consists of 9,970 M2 seed lots produced by INRA Rennes and stored under controlled conditions (5% RH, 8°C).

For DNA extractions, four seeds for each of the RAPTILL M2 seed lots were sown in individual pots. Leaf material was collected on 3-to-4-week old plants as a mixture of 16 leaf discs (Ø = 5 mm) per M2 family. After sample freeze-drying and grinding, DNA extractions were performed with the DNeasy 96 Plant Kit following the manufacturer's instructions (Qiagen, Chatsworth, CA, USA) and then tested for quality and quantity.

### FANCM homologues identification—screening of the BAC libraries

FANCM homologues were identified using reciprocal BLASTp and PSI-BLAST against the published *B. napus* (http://www.genoscope.cns.fr/blat-server/cgi-bin/colza/webBlat; Chalhoub et al., 2014), *B. rapa* (http://brassicadb.org/brad/blastPage.php; Wang et al., 2011) and *B. oleracea* (http://plants.ensembl.org/Multi/Tools/Blast?db=core) assemblies. Screening of the *B. napus* cv “*Darmor-bzh*” BAC library was performed by the CNRGV (INRA Toulouse) as described in (Lloyd et al., 2014).

### Search for mutations in *FANCM*—TILLING experiment

For *Brassica rapa*, we searched for mutations in *BraA.FANCM* (and *BraA.MSH4*) in the EMS mutagenized population of *B. rapa* subsp. *trilocularis* (Yellow Sarson) developed by the John Innes Centre (Stephenson et al., 2010; work conducted by Fran Robson at RevGenUK). Above and hereafter, we used a nomenclature adapted from Ostergaard and King (2008) where “categories are listed in descending order of significance from left to right (i.e., genus—species—genome—gene name)”: e.g., *Bra* stands for *B. rapa* while *Bna* stands for *B. napus*, A, and C are the two genomes where we searched for mutations in *FANCM* or *MSH4*, respectively.

In *B. napus*, two separate screens were carried out to find mutations affecting specifically *BnaA.FANCM* or *BnaC.FANCM* in a subset of 500 M2 plants from the RAPTILL population described above. These screens were based on the use of copy-specific primer pairs (Supplementary Figure S1) and implemented the PMM (Plant Mutated on its Metabolites) method (Triques et al., 2007, 2008; work conducted by Julien Schmidt at AELRED).

In both cases, TILLING targeted a region of 1Kb in the bipartite helicase domain of FANCM (Supplementary Figure S1). The list of the primers used for amplifying these regions is given in Supplementary Table S1. The primers were designed to amplify a single locus, i.e., they are copy-specific. We ensured that only one of the two homoeologous copies of FANCM was amplified in *B. napus* (Supplementary Figure S1).

### Plant material to evaluate the role of *FANCM* in *Brassica napus*

We initially selected three mutant alleles for *FANCM* in *B. napus* cv. *Tanto*: one nonsense mutation for the A copy (thereafter referred as to *bnaA.fancm-1*) and two missense mutations for the C copy (*bnaC.fancm-1* and *bnaC.fancm-2*). These mutations have a SIFT (Sorting Intolerant From Tolerant) score equal to zero, i.e., are predicted to be damaging to the protein (Sim et al., 2012). Two F1 hybrids combining *bnaA.fancm-1* with either *bnaC.fancm-1* or *bnaC.fancm-2* were first produced (*h1* and *h2* in Supplementary Figure S2). These F1s were then selfed to produce a full set of segregating F2 plants, among which we sought for plants homozygous for the two mutations (thereafter referred as double A/C mutants) or for the two WT alleles (thereafter referred as to WT siblings).

Two double A/C mutants (*bna.fancm_1-1*; which combined mutant alleles *bnaA.fancm-1* and *bnaC.fancm-1*) and two WT siblings (*Bna.FANCM_1-1)* were first identified in the progeny of the first F1 hybrid (*h1*; Supplementary Figure S2). These four plants, together with two single mutants homozygous for either *bnaA.fancm-1* or *bnaC.fancm-1*, were sequenced in order to identify in one go: (1) background EMS-induced mutations that can be used to develop Cleavage Amplified Polymorphism (CAPs) markers and (2) pairs of heterozygous intervals shared between mutant and WT F2s that can be used to compare crossover frequencies (Supplementary Figure S3). It turned out, however, that the *bna.fancm_1-1* mutants had no detectable effect on crossover formation (data not shown); these plants were therefore discarded for further analyses.

Two double A/C mutants (*bna.fancm_1-2*; which combined mutant alleles *bnaA.fancm-1* and *bnaC.fancm-2*) and two WT siblings (*Bna.FANCM_1-2*) were identified in the progeny of the second F1 (*h2*, Supplementary Figure S2). These plants were selfed to produce F3 progenies from which crossover frequencies were estimated genetically (Supplementary Figure S3). In the meantime, the F1 plant (*h2*), along with four other F1 hybrids combining *bnaA.fancm-1* with *bnaC.fancm-2*, were used to produce segregating populations of allohaploids following the protocol described in Jenczewski et al. (2003). For each F1 hybrid, 20–140 plants were regenerated through microspore culture; 20–40 allohaploid plants were then selected (per F1 hybrid) after validation of their ploidy level by flow cytometry. Molecular screening for *FANCM* alleles revealed the expected segregation pattern for the mutations, with 25% WT and 25% double (A/C) mutant allohaploids. For each F1 hybrid, a minimum of two double mutants and two WT siblings were selected for cytological evaluation (Supplementary Figure S2). This assay therefore encompassed two layers of replication: (1) the F1 hybrids that we used to derive allohaploids, each containing a different patchwork of background mutations inherited from the *bnaA.fancm-1* and *bnaC.fancm-2* parents and (2) the different double (A/C) *fancm* mutant and WT plants that were derived from a given F1 hybrid, each containing different combinations of mutations present at the heterozygous stage in the F1 (Supplementary Figure S3).

The different *fancm* mutations were detected using Cleaved Amplified Polymorphic Sequences (CAPS) assay targeting the causative EMS-SNP. The list of primers and restriction enzymes in given in Supplementary Tables S1, S2.

### DNA sequencing to identify background mutations

Total DNA was extracted using the NucleoSpin® Plant II Midi/Maxi (Macherey-Nagel) extraction kit. DNA sequencing was carried out by EPGV group (INRA, Evry). Whole genome libraries were prepared using the TruSeq® DNA PCR-Free LT kit (Illumina). Briefly, sample preparation was performed with the low sample protocol using a 550 bp fragment sizing; all enzymatic steps and clean-up procedures were performed according to manufacturer's instructions. The resulting indexed libraries, including the ligated adapter sequences, had a mean size of 870 bp. Clustering and pair-end sequencing (2 × 100 sequencing by synthesis (SBS) cycles) were performed in high output mode on a HiSeq® 2,000/2,500 (Illumina) according to manufacturer's instructions. The two single homozygous mutants for *bnaA.fancm-1* and *bnaC.fancm-1* were sequenced on the same single lane while the corresponding double homozygous and WT F2s were sequenced on a single lane each. Raw short-read data are available in the NCBI BioProject PRJNA432890.

Mutations were identified and annotated using the “homemade” pipeline MutDetect described in Girard et al. (2014). Briefly, sequences were aligned against the reference *B. napus* genome sequence (Chalhoub et al., 2014) allowing up to 2 mismatches and 1 indel per read. Alignments were cleaned up according to the Genome Analysis ToolKit (GATK) recommendations (McKenna et al., 2010). Raw variants were then filtered according to both quality and coverage criterions (quality> 100 and Depth>2). Homozygous variants detected on all samples were considered as natural polymorphisms between *Darmor-bzh* (reference) and *Tanto* accessions and were removed.

### Genetic assessment of co rate and variation

We used the sequence data obtained with the *bna.fancm_1-1* and *Bna.FANCM_1-1* plants to develop CAPS markers. These markers were used to genotype the *bna.fancm_1-2* and *Bna.FANCM_1-2* plants and identify pairs of heterozygous intervals shared between mutant and WT F2s (Supplementary Figure S2). This approach was constrained by the fact that the F2 plants we sequenced (the first we obtained) were not the same as the ones we used for this genetic assay. Consequently, many of the CAPS markers we developed failed to identify intervals that were heterozygous in both the mutant and WT F2s.

The list of primer pairs and restriction enzymes that we eventually used for genotyping F3 progenies is given in Supplementary Table S3. Crossover frequencies were estimated using MapDisto (Lorieux, 2012). The statistical significance of the pairwise difference between WT and mutant crossover frequencies was obtained using the Welch test with a significance threshold of 5% (Bauer et al., 2013).

### Cytology

Florets were fixed in Carnoy's fixative (absolute ethanol:acetic acid, 3:1, v/v). CO frequencies were inferred from male meiotic spreads after staining with either DAPI (as described by Chelysheva et al., 2013) or Acetocarmine (as described by Jenczewski et al., 2003). In *B. rapa*, in which we mainly observed bivalents (i.e., pairs of homologous chromosomes bound by COs), we used the criteria established by Moran et al. (2001) to estimate the number of chiasmata: rod bivalents were considered to be bound by one single chiasma in one arm only, whereas ring bivalents were considered to have both arms bound by one chiasma. In *B. napus* allohaploids, we rather counted the number of univalents (i.e., chromosomes that failed to form crossovers), which were a majority and easy to score. In both cases, a minimum of 20 pollen mother cells was examined in each plant.

### Pyrosequencing

Pyrosequencing was performed on meiotic cDNA and on gDNA to check for amplification bias. The following primers were used for amplification and sequencing:

pFANCMR:TTTCGTTGGCTAAATCTTCTTCCT,
pFANCMF:ACGAAGCAAACAGAGAAGAAGACC,
pFANCMS:TCTTCTGCCAATTCATTA

Primer pairs have been designed with Pyromark Assay Design v2.0.1.15 and the pyrosequencing reaction has been performed with PyroMark Q24 v2.0.6 of QIAGEN®.

### Directed mutagenesis constructs, plant transformation, and plasmid constructs

A *AtFANCM* genomic fragment from *A. thaliana* was amplified that included 618 nucleotides before the ATG and 1,029 after the stop codon. The PCR product was cloned, by Gateway (Invitrogen) into the pDONR207 (Invitrogen) to create pENTR-FANCM, on which directed mutagenesis was performed using the Stratagene Quick-change Site-Directed Mutagenesis Kit. For plant transformation, LR reaction was performed with the binary vector pGWB1 (Nakagawa et al., 2007). The resulting binary vectors were transformed using the Agrobacterium-mediated floral dip method (Clough and Bent, 1998) on double homozygous mutant plant (*fancm*^−/−^*/msh5*^−/−^).

## Results

### *FANCM* is present in a single copy *Per Brassica* genome

**Figure 1:**
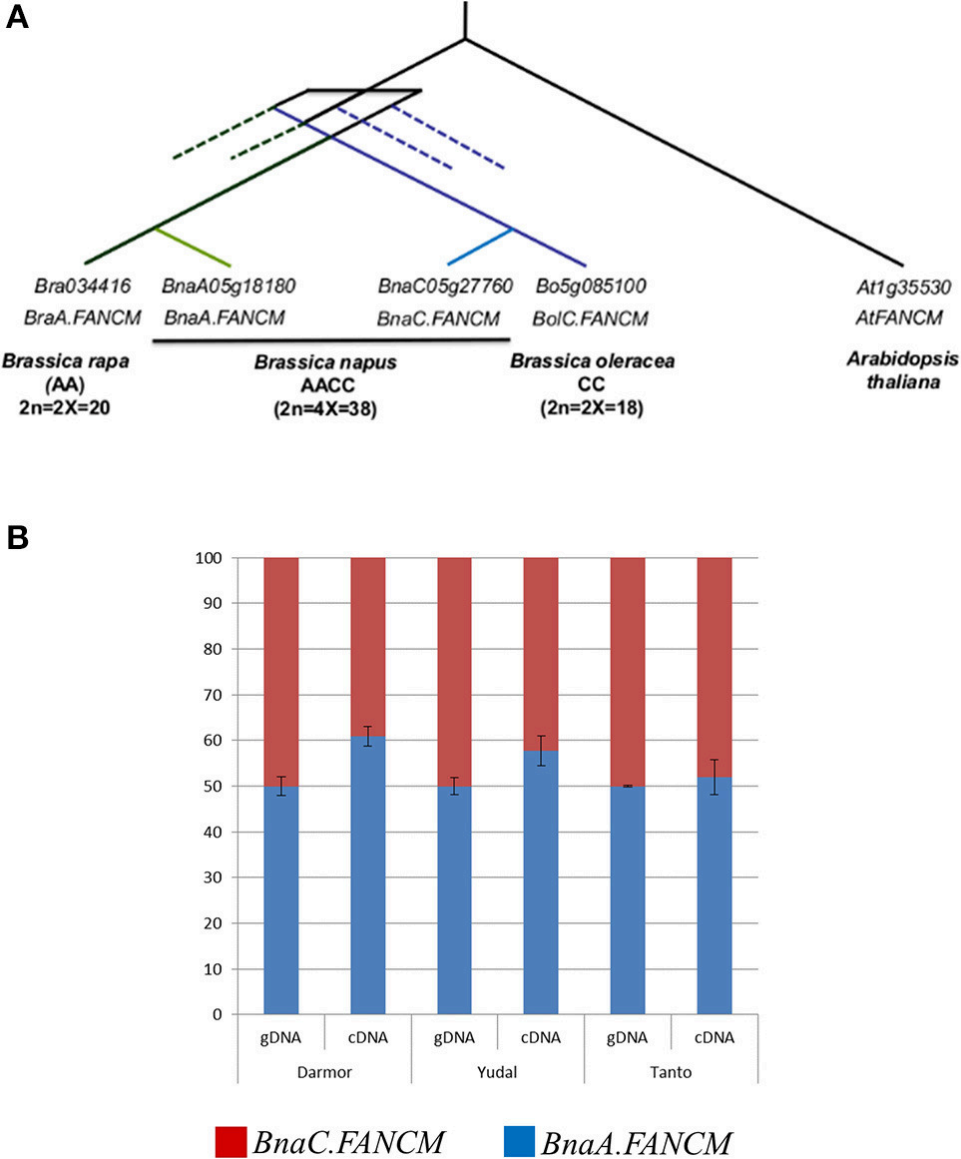
One copy of *FANCM* per genome is present and expressed in *Brassica napus*. **(A)** Schematic representation of the relationships between FANCM homologs in *Brassica rapa, B. napus, B. oleracea*, and *Arabidopsis thaliana*. Dotted lines represent the fractionated copies; i.e., copies originating from the Brassica specific whole genome triplication (WGT) and subsequently lost. **(B)** Relative contribution of *BnaA.FANCM* and *BnaC.FANCM* to total expression of *FANCM* in *Brassica napus*. Pyrosequencing data for *BnaA.FANCM* (blue) and *BnaC.FANCM* (red) in three varieties of *B. napus*. Genomic DNA (gDNA) was used as a control for biased PCR amplification between the two copies. Error bars = 1 SD from 3 biological replicates.

We first assessed the number of copies of *FANCM* that were retained in each Brassica genome following WGT. Querying the CDS of *At.FANCM* (JQ278026) against the available genome sequences revealed that *FANCM* has one single homologue in both *B. rapa* (*Bra034416* on chromosome A05, hereinafter referred as to *BraA.FANCM*) and *B. oleracea* (*Bo5g085100* on chromosome C05; *BolC.FANCM*) while *B. napus* contains two copies of *FANCM* (Figure 1A). The presence of two *FANCM* homologues in *B. napus* (*BnaA05g18180D* / *BnaA.FANCM* on A05 and *BnaC05g27760D*/*BnaC.FANCM* on C05) was further confirmed by BAC screening and sequencing. The sequences obtained from the BACs were instrumental to complete the full-length sequences of *BnaA.FANCM* and *BnaC.FANCM* that were still pending in the published assembly. These two genes are located within syntenic regions (Supplementary Figure S4) and form a pair of homoeologues. We used mRNA-Seq data produced from *B. napus* male meiocytes (Lloyd et al., 2018) to show that *BnaA.FANCM* and *BnaC.FANCM* are almost equally transcribed during meiosis in this species; this result was subsequently confirmed by pyrosequencing (Figure 1B).

We also used the mRNA-Seq data to confirm the sequence of *BnaFANCM* open reading frames. *BnaA.FANCM* and *BnaC.FANCM* have almost the same intron/exon structure; they only differ by the presence of a small (70 bp) additional intron in *BnaC.FANCM* (and *BolC.FANCM*) that splits Exon 2 but does not alter the final amino acid sequence. The two predicted proteins (based on the full-length cDNA sequences derived from the mRNA-Seq data) share >97% identity across their full length. They are highly related to At.FANCM (~81% identity and ~84% similarity with JQ278026), in particular in the regions of the DEXDc and a HELICc helicase domains (Supplementary Figure S5).

### EMS mutagenesis yielded point mutations predicted to alter the function of FANCM in *Brassica*

Two EMS (Ethylmethanesulfonate) mutagenized populations (one for *B. rapa* and one for *B. napus*; ~500 M2 plants each) were screened for mutations within ~1 kb of the bipartite helicase domain of FANCM (Supplementary Figure S1) where many loss-of-function mutations have been found in *A. thaliana* (Crismani et al., 2012).

In total, >100 mutations were identified across the three *FANCM* genes with considerable gene-to-gene variation (Supplementary Figure S1); i.e., more than twice as many EMS mutations were found in *BnaA.FANCM* and *BnaC.FANCM* compared to *Bra.FANCM*. This reflected an average density of one mutation every 13 Kb in the *B. napus* mutagenized population compared to 1/31 Kb in the *B. rapa* population (1/60 Kb in Stephenson et al., 2010). Around 75% of these mutations (77/104) were synonymous substitutions or occurred in introns (Supplementary Figure S1) and only one non-sense mutation was identified among the three genes (in *BnaA.FANCM*). These estimates are similar to previous findings from M2 lines of the same (*B. rapa*) and other mutagenized populations (see Gilchrist et al., 2013 and ref. therein).

We retained the single non-sense mutation for *BnaA.FANCM* (hereinafter referred to as *bnaA.fancm-1)* identified in our screen; this mutation induced a premature stop codon between the DEXDc and the HELICc domain (Supplementary Figure S5). For the other two *FANCM* genes, we selected missense mutations altering amino acids conserved across representative eukaryotes species and predicted to be damaging to the protein (SIFT score = 0.00). For *BraA.FANCM*, one missense mutation (hereinafter referred as to *braA.fancm-1)* was retained, which consisted of a substitution of a proline at position 443 for a leucine (Supplementary Figure S5). For *BnaC.FANCM*, two missense mutations (hereinafter referred to as *bnaC.fancm-1* and *bnaC.fancm-2*) were selected; *bnaC.fancm-1* consisted of a leucine to phenylalanine substitution at position 330 while *bnaC.fancm-2* consisted of a glycine to arginine substitution at position 393 (Supplementary Figure S5). Interestingly, substitution of the same glycine for glutamic acid was shown to be causal for a defective FANCM protein in *A. thaliana* (Crismani et al., 2012).

### FANCM limits Co frequencies in *Brassica rapa*

To test whether FANCM limits COs in *B. rapa*, we replicated the cytological assay that was used to first identify the anti-CO activity of this protein in *A. thaliana* (Crismani et al., 2012); i.e., we tested whether *bra.fancm-1* was able to restore bivalent formation in a class I CO-defective mutant.

For this, we first identified through TILLING a deleterious mutation in *BraA.MSH4* (hereinafter referred as to *braA.msh4-*1; Supplementary Figure S6), the single copy homologue of *AtMSH4*- an essential ZMM protein (Higgins et al., 2004) *-* in *B. rapa* (Lloyd et al., 2014). The mutation *braA.msh4-1* induced a substitution in the acceptor site of the 19th exon (*BraA.MSH4* has 24 exons) right after position 626; this introduced a premature STOP codon in the predicted coding sequence of the essential MutS domain of the MSH4 protein (Obmolova et al., 2000; Higgins et al., 2004; Nishant et al., 2010; Wang et al., 2016).

**Figure 2:**
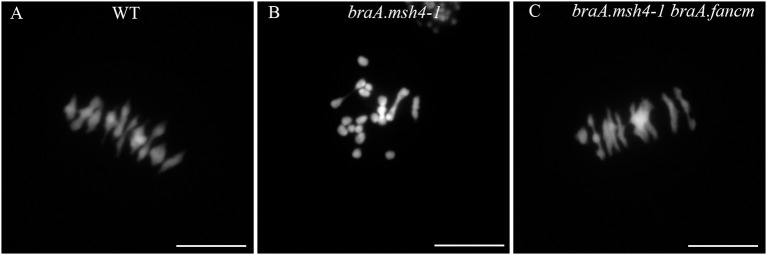
Restoration of bivalent formation in the double mutant *braA.msh4-1*^−/−^ *braA.fancm-*^−/−^ **(A)** During metaphase I in WT *B. rapa*, 10 bivalents and no univalent are formed. They are all aligned on the metaphase plate. **(B)** In the single *braA.msh4-1*^−/−^ mutant, only a few bivalents are formed, most of the chromosomes remain as univalents. **(C)** Metaphase I in the double mutant *braA.msh4-1*^−/−^ *braA.fancm-*^−/−^ is reminiscent of metaphase I in WT *B. rapa*, mostly bivalents are formed, only ~0.5 univalent pair is found on average per cell. Scale bar = 10 μm.

We first confirmed that *braA.msh4-1* resulted in a significant shortage in CO formation in *B. rapa*. Plants homozygous for *braA.msh4-1* (*braA.msh4-1*^−/−^) showed a mixture of bivalents (3.7 ± 1.4 per cell; *n* = 43 cells) and univalents (6.3 ± 1.5 per cell) at metaphase I when WT plants systematically formed 10 bivalents (*n* = 66) (*p*-value = 6.16 E-30; Figures 2A,B). Coupled with this reduction in bivalent formation was a difference in the shape of the bivalents. While 50% of the bivalents in WT were rings with both arms bound by chiasmata, 88% of the bivalents in *braA.msh4-1*^−/−^ were rods with only one arm bound by chiasmata (Figures 2A,B). Assuming that rod and ring bivalents had only one and two COs, respectively, we estimated that the mean number of COs dropped significantly from 14.8 ± 1.5 COs per cell (*n* = 36) in WT to 4.05 ± 1.82 COs per cell (*n* = 40) in *braA.msh4-1*^−/−^ (*p*-value = 9.2 E-44). These observations are reminiscent of the meiotic behavior of *Atmsh4* single mutant (Higgins et al., 2004).

We then produced a plant containing mutations in both the *BraA.MSH4* and *BraA.FANCM* genes (*braA.msh4-1*^−/−^ *braA.fancm-1*^−/−^) and assessed meiotic crossover frequency in this double mutant using the same cytological approaches. We observed a large increase in bivalent formation (9.4 ± 0.7 bivalents; *n* = 66) compared to *braA.msh4-1*^−/−^ (*p*-value = 1.8 E-33; Figures 2B,C). However, the number of bivalents in the double mutant *braA.msh4-1*^−/−^ *braA.fancm-1*^−/−^ remained significantly different from that of the WT (unpaired *t*-test; *p*-value < 0.0001) due to the presence of a small number of univalents (0.57 univalent per cell in *braA.msh4-1*^−/−^ *braA.fancm-1*^−/−^; Supplementary Figure S7). This observation indicated a random distribution of CO consistent with the absence of obligate class I COs (Crismani et al., 2012). We also observed that ~50% of bivalents in *braA.msh4-1*^−/−^ *braA.fancm1*^−/−^ were rings and estimated that the mean chiasma frequency in this plant (14.0 ± 2.9 per cell; *n* = 35) was indistinguishable from that observed in WTs. This represented an increase of at least 10 COs in *braA.msh4-1*^−/−^ *braA.fancm-1*^−/−^ compared to the single mutant *braA.msh4-1*^−/−^. Bearing in mind that it is not possible to distinguish cytologically single from multiple COs clustered on a single arm (Supplementary Figure S7), this increase probably underestimates the extent to which FANCM shut down CO frequency in *B. rapa*. This notwithstanding, our results clearly demonstrate that BraA.FANCM, like At.FANCM, limits CO formation in *msh4* mutants.

### *FANCM* limits homologous crossovers in *Brassica napus*

Replicating the same cytological assay in *B. napus* was not feasible, due to the lack of *msh4* mutants in this species at the time of the study. We therefore developed a genetic assay to assess the effect of FANCM on crossover frequency in *B. napus*. This approach took advantage of the fact that plants defective for either *BnaA.FANCM* or *BnaC.FANCM* had to be crossed to produce a loss-of-function *fancm* double mutant in *B. napus* (Supplementary Figure S2). Given the EMS-mutation density observed within *BnaA.FANCM* and *BnaC.FANCM*, we reasoned that these (F1) hybrids contained an extensive set of EMS mutations that could be used as a source of polymorphism for subsequent genetic analyses (Supplementary Figure S3).

To identify these segregating mutations, we sequenced two *bna.fancm_1-1* mutants and two of their WT siblings (*Bna.FANCM_1-1*) (Supplementary Figure S2). Sequencing quality control process showed that around 70% of the reads were mapped (Supplementary Table S4) covering around 80 % of the genome reference with a minimum depth of 3x (Supplementary Figure S8). Using conservative criteria, we detected ~20 763 EMS mutations in the sequenced plants (Supplementary Table S4; Supplementary dataset 1), which was consistent with mutation density within *BnaA.FANCM* and *BnaC.FANCM*. ~23 % (4714/20763) of those mutations were found in CDS and led to non-synonymous substitutions (including splice variant and non-sense mutations) in a total of 4,438 genes (~4% of total gene number; Supplementary dataset 2). A subset of those genes (270 genes with 298 mutations; ~8%) constituted homoeologous pairs (as established in Chalhoub et al., 2014); in most cases (193/298, 64%), the mutations that we found in both copies of a given homoeologous pair were non synonymous or stop gained mutations (Supplementary dataset 2). None of these EMS mutations were found in the orthologs of genes that encode most other known anti-CO proteins (e.g., MHF1, MHF2, FIGL1, FLIP, RMI1, TOP3α, RECQ4a/b) in *A. thaliana* (Supplementary Table S5). Finally, overall all four sequenced plants, only 66 mutations targeting 30 homoeologous pairs were detected in the homozygous state at both loci in at least one plant. The risk of confusion between the effect of *fancm* mutations and that of another pair of homoeolog is thus very low.

We then converted a subset of the EMS induced SNPs into genetic markers and compared crossover frequencies between mutant and WT F2 plants in the corresponding F3 progeny (Table 1; Supplementary dataset 3). For the three intervals examined, which were all located in the most distal part of the chromosomes where CO frequencies are the highest (Lloyd et al., 2018), we observed a significant increase in crossover frequency (~32%, Welch's *t*-test; *p*-value < 0.013) in the progeny of *fancm* mutants compared to the progeny of WT plants (Table 1). Altogether, the consistency of the increase in crossover frequency observed for three intervals suggests that FANCM limits crossover formation in *B. napus*. However, as this increase in COs was rather limited compared to what was observed in *B. rapa* mutants, our results cast doubt as to whether the *bnaC.fancm-2* mutation resulted in complete loss of FANCM anti-crossover activity (see below).

**Table 1:** Homologous crossover frequencies in homozygous WT and mutant for *FANCM* in *Brassica napus*.

**Chromosome**	**Interval^a^**	**Physical size (Mb)**	**Observed genetic size in WT^b^**	**Observed genetic size in *fancm* mutant^a^**	**Fold-change**	***p*-value^c^**
A01	1	1.611	19.1 cM (*n* = 101) [18.4–19.8]	24.3 (*n* = 131) [23.6–24.9]	1.3	0.011
C01	2	0.575	5.4 (*n* = 96) [5.0–5.7]	7.2 (*n* = 102) [6.8–7.5]	1.3	0.002
A05	3	1.135	11.3 (*n* = 86)^d^ [10.60–11.8]	14.4 (*n* = 134) [14.0–14.8]	1.3	0.006

a All these intervals are located in the most distal part of the chromosomes — see Supplementary Table S3 for detailed positions.

b The number of plants genotyped per progeny is given (in parentheses). Confidence intervals (mean ± 1.96 × SE) are given [in square brackets].

c Considering a one-tailed hypothesis: i.e., crossovers in fancm mutant > crossovers in WT.

d *This distance was estimated using the F3 progeny of Bna.FANCM_1-1 due to lack of polymorphim in Bna.FANCM_1-2*.

### *FANCM* limits Co formation in *Brassica napus* allohaploids

In *B. napus*, microspore culture can be used to produce allohaploid plants (AC) that contain one unique copy of each of the 19 *B. napus* chromosomes (*n* = 19) and thus no longer have homologous chromosomes. We previously reported that meiotic crossovers can readily form between homoeologous chromosomes in these plants (Grandont et al., 2014). This suggests that the recombination intermediates upon which FANCM could potentially act may also exist in *B. napus* allohaploids. We thus derived allohaploid progeny from five different plants, each combining the *bnaA.fancm-1* and *bnaC.fancm-2* mutations at the heterozygous state (Supplementary Figure S2). In each of these progenies, two to five *fancm* (A/C) double mutant and *FANCM* WT plants were recovered and used to compare CO frequencies using cytological approaches (Supplementary Figure S2).

**Figure 3:**
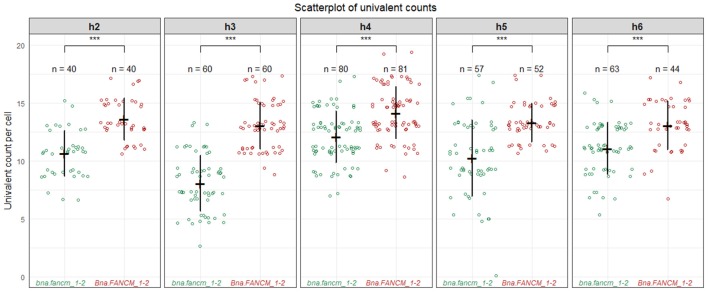
Homoeologous crossovers in *fancm* allohaploids plants. Boxplot for the number of univalents between mutants and WT allohaploids derived from five heterozygous F1 plants combining *bnaA.fancm-1* with *bnaC.fancm-2* (h2–h6). At least 2 mutants and 2 WT allohaploids plants were derived per F1 hybrids and were used as replicates. Around 20 meiocytes have been observed per replicate and the counts per replicate were pooled together. ***P < 0.001, Wilcoxon Signed-Rank Test.

We first observed that WT allohaploid plants showed a low number of bivalents (2.8 ± 1.3 per cell; *n* = 59 cells) and a majority of univalents (13.4 ± 2.7 per cell) at metaphase I. *Tanto* is therefore among the varieties that form little CO at the allohaploid stage (see Grandont et al., 2014) We were thus best positioned to detect a small increase in COs, if any. This is exactly what we observed: i.e., a significant and consistent increase in bivalent formation when comparing *bna.fancm_1-2* mutant allohaploid and *Bna.FANCM_1-2* WT allohaploid plants. The mean number of chromosomes that failed to form a CO decreased from 13.5 in WT (71%) to 10.5 in *fancm* (55%) (Wilcoxon signed rank test, *p*-value = 0.0016). This trend was observed for all allohaploids and all F1 hybrids, with some variation in the magnitude but no variation in the direction of the change (Figure 3). We believe this is unlikely to be a mere coincidence. The systematic correspondence observed between double (A/C) *fancm* mutants and increased CO frequencies across all F1s and all allohaploids rather suggests that FANCM limits CO formation between homoeologous chromosomes in *B. napus*.

### BnaC.FANCM-2 has still residual Anti-CO activity

Given the small but significant increase in CO frequency repeatedly observed in *bna.fancm_1-2* mutant plants, we assessed whether the protein encoded by *bnaC.fancm-2* (thereafter BnaC.FANCM-2) has still some anti-CO activity. We reasoned that *bnaA.fancm-1*, which induced a premature stop codon between the DEXDc and the HELICc domain, is likely a loss-of-function mutation and thus only questioned the effect of *bnaC.fancm-2* on CO formation.

**Figure 4:**
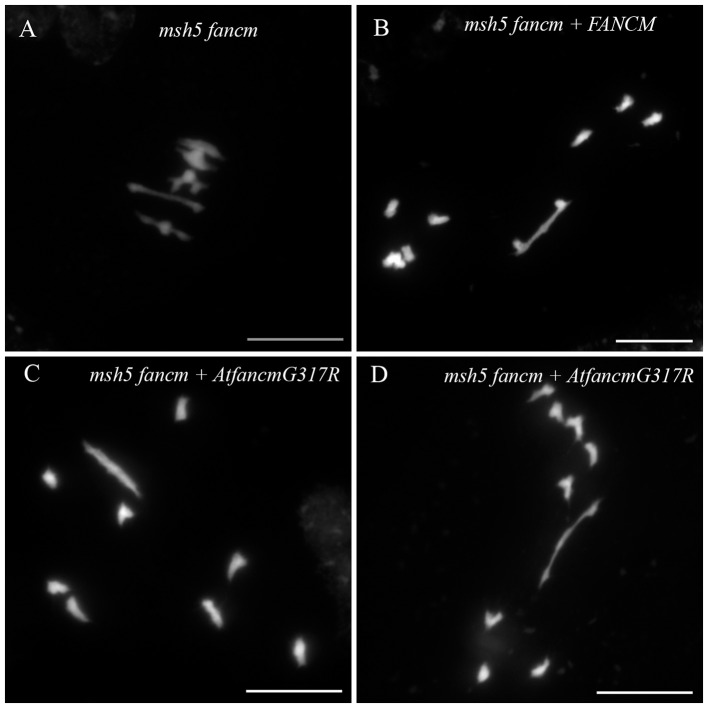
Bivalent formation in *A. thaliana msh5 fancm* double-mutant transformed with different version of *FANCM*. During metaphase I, 5 bivalents were observed in *msh5 fancm* double-mutant meiocytes **(A)**. When complementing *msh5 fancm* with the WT allele of *At_FANCM* **(B)**, or with a modified copy of *FANCM* (*AtfancmG317R*) mimicking *BnaC.fancm-2* **(C,D)**, mainly univalents were observed. Scale bar = 10 μm.

In order to do so, we transformed an *A. thaliana msh5 fancm* double-mutant with a modified copy of *At.FANCM* that lead to the same amino acid substitution found in *BnaC.fancm-2*. The *A. thaliana msh5 fancm* double-mutant was fertile and displayed ~5 bivalents per cell (*n* = 14) as in the WT (Figure 4, Table 2). We reasoned that the number of bivalents should remain essentially the same in the transformant if the transgene encodes a completely non-functional protein. On the contrary, the transformant should demonstrate a decay in bivalent formation if the transgene encodes a (partially) functional protein. We tested these predictions by transforming the *msh5 fancm* double-mutant with the WT allele of *At.FANCM*. As expected, the number of bivalents in the transformant dropped down to that observed in *Arabidopsis msh5* mutant (~1.5 bivalent per cell, *n* = 12; Figure 4, Table 2). We observed essentially the same pattern with the transgene mimicking *BnaC.fancm-2*: numerous univalents, a mean number of ~1.5 bivalents (*n* = 17) bivalents and clear evidence of unbalanced chromosome segregation after meiosis I (Figure 4, Table 2). These results indicate that BnaC.FANCM-2 retained anti-CO activity. It is worthy of note, however, that the endogenous level of BnaC.FANCM-2's residual activity in *bna.fancm_1-2* mutant plants can hardly be extrapolated from this experiment.

**Table 2:** Chromosome segregation in *A. thaliana msh5 fancm* double-mutant transformed with different version of *FANCM*.

	**Number of bivalents**	**0**	**1**	**2**	**3**	**4**	**5**
*msh5^−/−^fancm^−/−^*		0	0	0	0	1	13
*msh5^−/−^fancm^−/−^*	+ *AtFANCM*	0	6	4	2	0	0
*msh5^−/−^fancm^−/−^*	+ *Atfancm-G404R*^a^	2	9	4	1	1	0

a *modified version of AtFANCM mimicking bnaC.fancm-2, i.e., containing a glycine to arginine substitution at position 404*.

## Discussion

Identification of genes encoding anti-CO proteins in *A. thaliana* holds great promise to improve the efficiency of plant-breeding programs (Crismani et al., 2013). In this study, we combined BAC screening, TILLING, whole-genome resequencing, cytology, genotyping and complementation tests (in *Arabidopsis*) to demonstrate that FANCM limits COs in two Brassica crops. To the best of our knowledge, this is the first example of a translational biology approach to increase CO frequencies in crops (see Mieulet et al., 2016 on a related, yet different topic).

### FANCM limits crossovers in *Brassica* crops

Altogether our results indicate that the anti-CO activity of FANCM is conserved in two important *Brassica* crops, thus probably across the entire *Brassicaceae* family. This point is more strikingly illustrated in *B. rapa* where we observed a strong increase in COs in the *fancm/msh4* double mutant compared with the single *msh4*. This change is consistent with the 3-fold increase in COs reported in *A. thaliana* (Crismani et al., 2012); like Arabidopsis, the extra COs were sufficient to restore bivalent formation to a WT level in *B. rapa* (Figure 2).

A less pronounced increase in CO frequency (~1.3-fold) was observed in *B. napus* (Table 1), probably because the amino acid substitution found in our *B. napus bnaC.fancm-2* mutant allele does not completely ablate FANCM's anti-CO activity (Figure 4). In spite of this residual anti-CO activity, we repeatedly observed a small but significant increase of: (i) crossover frequencies across three independent genetic intervals in euploids (Table 1) and (ii) bivalent formation across all biological replicates in allohaploids (Figure 3) produced from *BnaA.FANCM-1*^+/−^*BnaC.FANCM-2*^+/−^. These results lend support to the hypothesis that FANCM limits CO formation in *B. napus* too.

It is important to underline here that we don't know how sensitive the complementation test is. For example, it is uncertain whether expression of a transgene (in Arabidopsis) consisting in a modified version of the Arabidopsis WT allele of FANCM faithfully recapitulates the activity of the protein encoded by Brassica *BnaC.fancm-2* mutant allele expressed at its endogenous level and in *B. napus*. This approach could simply be too conservative to reveal the extent to which the substitution identified in *bnaC.fancm-2* is detrimental for FANCM anti-CO activity. Identification and/or production of loss-of-function mutations for *BnaC.FANCM* is thus required to determine how much CO frequency can be increased in *B. napus fancm* mutants.

### Does FANCM limit crossover formation between homoeologous chromosomes?

In *A. thaliana*, increased crossover frequency in *fancm* mutants is suppressed in heterozygous regions (Girard et al., 2015; Ziolkowski et al., 2015). It is thus surprising that we detected a small but repeatable effect of *fancm* mutations on CO formation in *B. napus* allohaploids (Figure 3) where crossovers are necessarily formed between homoeologous chromosomes. The SNP density between homoeologous transcripts (~3.5%; Cheung et al., 2009), which is a lower bound estimate of the overall rate of polymorphism between the A and C genomes, is indeed much higher than the allelic SNP diversity measured between *B. napus* varieties (~0.049–0.084%; Trick et al., 2009). It is also higher than the SNP density observed between *Arabidopsis* accessions (0.5%) (Alonso-Blanco et al., 2016). Our results may therefore suggest that the extra COs observed in *B. napus fancm* mutants are either less sensitive to heterozygosity than they are in *A. thaliana* or are actually not formed between homoeologous regions in *B. napus fancm* mutant allohaploids.

In *B. napus*, genomic exchanges between homoeologous chromosomes can eliminate polymorphism in some regions i.e., some homoeologous chromosomes contain homologous segments (Chalhoub et al., 2014; He et al., 2017; Samans et al., 2017; Lloyd et al., 2018). Although homoeologous exchanges have yet to be characterized in the cultivar used in that study (cv. *Tanto*), there is no reason to believe that this variety would be an exception. All of the *B. napus* cultivars analyzed so far contain at least one and usually 10–12 homoeologous exchanges (Chalhoub et al., 2014; He et al., 2017; Samans et al., 2017). It is therefore tempting to hypothesize that the increase in bivalent formation observed in allohaploid *fancm* mutants results from increased CO formation within shared homologous regions on otherwise homoeologous chromosomes. This would represent a similar situation to that described by Ziolkowski et al. (2015), where juxtaposed heterozygous and homozygous regions biased the distribution of extra CO in *Arabidopsis fancm* mutants toward the homozygous intervals. Our hypothesis is also supported by the fact that bivalent formation between chromosomes 5D of wheat and 5M of *Aegilops geniculta* is promoted by pre-existing homoeologous exchanges; Koo et al. (2016) observed that >60% of the crossovers formed between 5D and 5M occurred in the terminal homologous part that is shared between the two chromosomes, even though this region only represents 5% of the physical length of those chromosomes.

Thus, the difference observed between *fancm* mutant and WT allohaploids could reflect a difference of homologous rather than homoeologous recombination. Testing this hypothesis would require assessing whether (i) the increase in CO frequencies occurs in very specific and usually small (Samans et al., 2017) chromosomal regions and (ii) these regions experienced homoeologous exchanges beforehand. This approach, which can theoretically be envisaged in *B. napus* (Howell et al., 2008), will first require homoeologous exchanges to be identified in cv. *Tanto*.

### Translational biology to increase crossover frequencies in crops

As reviewed by Wijnker and de Jong (2008), “meiotic recombination has a pivotal role in successful plant breeding.” Increasing crossover frequencies could notably generate new allelic combinations and a broader range of genotypes, decrease and slow down the loss of genetic variance during selection process, reduce linkage drag, facilitate a more efficient purging of mutation load… These opportunities are now within reach (Fernandes et al., 2017), provided that basic research on meiotic recombination is translated into crops. Our results show that this is possible, paving the way for further studies in other crops and/or with other antiCO proteins.

Translating the knowledge gained in *A. thaliana* into cultivated species, e.g., producing hyper-recombinant crop plants, supposes first to knock-out the homologs for genes encoding antiCO proteins in crops. For many species, the most common approach remains TILLING. In our study, we successfully applied TILLING to find mutations across three *FANCM* genes in *Brassica*. Our results clearly demonstrate that the effect of missense mutations on protein function is difficult to predict, even if these mutations target highly conserved amino acids located in essential domains. This observation, which is not specific to our study (Kumar et al., 2009), is well illustrated by the mutation *BnaC.fancm-*2. As described above, this missense mutation did not completely abolish BnaC.FANCM anti-CO activity (Figure 4) while it altered the same glycine that was shown to be essential for FANCM anti-CO activity in *A. thaliana* (Crismani et al., 2012). Looking to the future, new methodologies that increase the chance of finding nonsense mutations should be favored. This may involve the use of next-generation sequencing to “enable a deep search for mutations in targeted loci” (Tsai et al., 2011; see also Gilchrist et al., 2013) or the development of sequenced mutant populations (Krasileva et al., 2017; see also http://revgenuk.jic.ac.uk/search-databases/ for *B. rapa*). As a matter of fact, screening the RAPTILL population for mutations along the entire coding sequence of *BnaC.FANCM* would have reduced the chances of detecting no nonsense mutations from ~42 to ~3.5%.

However efficient the new TILLING approaches may be, it remains that the use of highly mutagenized populations raises concern about the risk of mistaking the consequences of a background EMS mutation for a mutation in the targeted gene. While it is necessary to control that risk, attempts to purge the mutation load off through backcrossing would be both inefficient and ineffective. In *B. napus*, given the density of EMS mutations we disclosed in this study, we estimated that >1,000 EMS mutations would still be segregating after 3 backcrosses to the WT (i.e., ~2 years). In the context of translational biology, verifying how strong is the correlation between the presence of the mutation in the target and the phenotype of biological replicates (siblings mutant plants harboring different combinations of EMS background mutations) constitutes a more reasonable approach. When possible, the use of series of allelic mutations can also be used to demonstrate the causal relationship between these mutations and the phenotype (Stephenson et al., 2010).

It is however important to note that we searched for a very specific and uncommon phenotype (increase of CO frequencies) to which only a few genes have been shown to contribute. Thus, the risk of confounding the effect of background mutations with the targeted mutant alleles should not be overestimated. In this regards, we verified that the plants we sequenced were free of mutations in the orthologs of *MHF1, MHF2, FIGL1, FLIP, RMI1, TOP3*α, or *RECQ4ab* (Girard et al., 2014, 2015; Séguéla-Arnaud et al., 2015; Fernandes et al., in review) (Supplementary Table S5). The possibility remains, however, that new anti-CO factors could have been targeted, which have yet to be identified in Arabidopsis or in another model plant (Hu et al., 2017).

In addition, we showed here that the chance that a *fancm* mutant plant also contains deleterious mutations affecting another pair of homoeologous genes (each at the homozygous stage) is very low. As expected, this probability peaks in the vicinity of *Bna.FANCM* genes (on chromosome A05 and C05), due to linkage drag (Supplementary Figure S9). Because each of the F2 plants contains a different patchwork of background EMS mutations due to independent segregation, the chance of finding such events shared between two F2 mutants or a F2 mutant and a WT sibling is even lower. We found only 4 such events in our assay, 2 mutations in common between two F2 mutants and 2 mutations in common between the 2 wild type siblings.

The rapid development of the CRISPR-CAS9 technology offers new opportunities to target mutagenesis and circumvent the off-target mutations issue in many crops (Brooks et al., 2014; Wang et al., 2014; Li J. et al., 2016), including *B. napus* (Braatz et al., 2017; Yang et al., 2017). The ability of CRISPR-CAS9 to simultaneously generate stable and heritable mutations in the different homoeologous copies of a gene, opens new avenues for future translational research. For example, it makes it possible to test whether, like in *A. thaliana*, meiotic crossover is unleashed in plants defective for multiple antiCO proteins (Séguéla-Arnaud et al., 2015; Fernandes et al., 2017). The challenge of producing and characterizing hyper-recombinant plants should not however overshadow the need for strategies aimed at maximizing the benefits of increasing CO frequencies in crops. In order to be adopted, the use of hyper-recombinant plants must fit into the framework of the current breeding schemes. In that regard, the fact that all causal mutations conferring increased CO frequency are recessive constitutes a limitation. Both methodological (e.g., breeding strategies) and biotechnological (e.g., dominant systems) developments will therefore be needed before “engineered meiotic recombination” becomes part of plant breeders' arsenal.

## Author contributions

EJ, AL, and A-MC designed the research. ABlary, AG, FE, ABérard, HB, NB, DC, CC, LC, JF, CG, M-CLP, ML, M-OL, NN, and AL performed the research. ABlary, AG, A-MC, and EJ analyzed the data. ABlary, AG, and EJ wrote the paper.

### Conflict of interest statement

The authors declare that the research was conducted in the absence of any commercial or financial relationships that could be construed as a potential conflict of interest.
