# Corrigendum: FANCM Limits Meiotic Crossovers in Brassica Crops

**DOI:** 10.3389/fpls.2020.604728

**Published:** 2020-12-04

**Authors:** Aurélien Blary, Adrián Gonzalo, Frédérique Eber, Aurélie Bérard, Hélène Bergès, Nadia Bessoltane, Delphine Charif, Catherine Charpentier, Laurence Cromer, Joelle Fourment, Camille Genevriez, Marie-Christine Le Paslier, Maryse Lodé, Marie-Odile Lucas, Nathalie Nesi, Andrew Lloyd, Anne-Marie Chèvre, Eric Jenczewski

**Affiliations:** ^1^Institut Jean-Pierre Bourgin, Institut National de la Recherche Agronomique, AgroParisTech, Centre National De La Recherche Scientifique, Université Paris-Saclay, Versailles, France; ^2^IGEPP, Institut National de la Recherche Agronomique, Agrocampus Ouest, Université de Rennes 1, Le Rheu, France; ^3^EPGV US 1279, Institut National de la Recherche Agronomique, CEA-IG-CNG, Université Paris-Saclay, Evry, France; ^4^Institut National de la Recherche Agronomique UPR 1258, Centre National des Ressources Génomiques Végétales, Castanet-Tolosan, France

**Keywords:** FANCM, Translational biology, *Brassica*, meiotic crossover, TILLING, plant breeding, polyploidy

We have noticed an error in the description of the mutation we used for BraA.FANCM.

In the original article, we stated that the mutation we retained for this gene (referred as to *braA.fancm-1*) consisted of a substitution of a proline at position 443 for a leucine. This was a mistake. In fact, *braA.fancm-1* consisted of a substitution of a glycine at position 497 for an aspartic acid. We have updated Supplementary Figure 5 accordingly.

The authors apologize for this error and state that this does not change the scientific conclusions of the article in any way. The original article has been updated.

