# Supplementary material for: FANCM Limits Meiotic Crossovers in Brassica Crops
Source: Front Plant Sci. 2018 Mar 23;9:368. doi: 10.3389/fpls.2018.00368 (PMC5876677; doi:10.3389/fpls.2018.00368)
Supplement: Supplementary file 4 [file DataSheet4.DOCX]

Supplementary Material

**FANCM limits meiotic crossovers in Brassica crops**

Aurélien Blary^a^, Adrián Gonzalo^a^, Frédérique Eber^b^, Aurélie Bérard^c^, Hélène Bergès^d^, Nadia Bessoltane^a^, Delphine Charif^a^, Catherine Charpentier^a^, Laurence Cromer^a^, Joelle Fourment^d^, Marie-Christine Le Paslier^c^, Maryse Lodé^b^, Marie-Odile Lucas^b^, Nathalie Nesi^b^, Andrew Lloyd^a^, Anne-Marie Chèvre^b^ & Eric Jenczewski^a, *^

*** Correspondence:** Dr. Eric Jenczewski; eric.jenczewski@ inra.fr

# Supplementary Data

**Supplementary dataset1:** Full genetic variation identified in the six sequenced plants (vcf files).

**Supplementary dataset2:** List of EMS-SNPS mutations predicted to affect protein function according to the SIFT algorithm

Given as a supplementary excel file

**Supplementary dataset3:** Genotyping data used to compute and compare recombination frequencies

Given as a supplementary excel file

# Supplementary Tables

**Supplementary Table S1**: List of primer pairs used to search for mutations through TILLING

| **Target Gene** | **Primer name** | **sequence** |
| --- | --- | --- |
| *BraA.FANCM* | Bra034416_F1 | GTGCTTCACGAAACTTATCCCTTGCCA |
|  | Bra034416_R2 | ggcataatccgataaaagtggcactgg |
| *BnaA.FANCM* | FANCMAF1 | CCAAAATGTGTTCCAAATTCATC |
|  | FANCMAR2 | GGGATGGTTTAAGAACAAATCATA |
| *BnaC.FANCM* | FANCMCF1 | CCAAAATGTGTTCCAAATTCATT |
|  | FANCMCR2 | GGGATGGTTTAAAAACAAATCAAG |
|  | Dcaps_FANCMC1F | CATTCGCAAGCTTCTTCCTAGTCAT |
|  | dcaps_FANCMC1R | TTGGACAATTTCGGGCTTGG |
| *BraA.MSH4* | MSH4F | ATGCAGAATGTCTCGAACCG |
|  | MSH4R | GCTTTGAGCGATAACAGATGC |

**Supplementary Table S2**: CAPS markers to identify the *fancm* and *msh4* mutant alleles in segregating populations

| **Target** | **Primer pairs ^1^** | **Restriction with** | **Products** | |
| --- | --- | --- | --- | --- |
|  |  |  | **WT allele** | **mutant** |
| *bnaA.fancm -1* | FANCMAF1-FANCMAR2 | XmnI/PdmI | 698bp+433bp | 1129bp |
| *bnaC.fancm -1* | FANCMCF1-FANCMCR2 | DraI | 1129bp | 972bp+157bp |
| *bnaC.fancm -2* | FANCMCF1-FANCMCR2  then  Dcaps_FANCMC1F-dcaps_FANCMC1R | BsiYI | 215bp+23bp | 238bp |
| *braA.msh4-1* | MSH4F – MSH4R | HpaII | 175bp+18bp | 196bp |

^1^: Primer sequences are given in Supplementary Table S1. A nested PCR is required to identify *bnaC.fancm -2*

**Supplementary Table S3**: List of primer pairs and restriction enzymes used in the genetic assay to measure CO frequencies between homologous chromosomes

| **Interval** | **Marker** | **Position^1^** | **Forward primer sequence** | **Cut** | **Cut** | **Product** | |
| --- | --- | --- | --- | --- | --- | --- | --- |
|  |  |  |  |  | **with** | **WT allele** | **mutant** |
| 1 | A01_426 | 77426 | TTGCATTGGTCCACACCCCAAGGAT | CAGGTTCAGCTTGCTCACAGGTGG | FokI | 50bp +  35bp | 85bp |
| 1 | A01_913 | 1688913 | ATGGGAACGACAGGACTGAG | TGTCCTCACCATCGGCTAAA | BseNI | 514bp | 371bp+143bp |
| 2 | C01_477 | 81477 | AAACACGAAAATTTAGAGAACCG | GTTTCAACGCTTCCCAATGC | AciI | 51bp +  23bp | 74bp |
| 2 | C01_152 | 657152 | CCAATGGGGTTTAATGGGCTC | AGACTCGAAAGGTTCCAGCA | SfaNI | 173bp + 134bp + 64bp | 198bp+173bp |
| 3 | A05_215 | 1012215 | ACATAACACATAACGGCAAATG | CACTGTTGGACCCGTCTATT | Hind III | 630bp + 31bp | 661bp |
| 3 | A05_252 | 2147252 | ACGACATCCAGTTTGCAGAT | GTGAAGTGGAAAATTTTAAGCATGA | MjaIV | 300bp + 188bp + 92bp + 64bp | 364bp + 188bp + 92bp |

^1^: this is the distance from the very beginning of the pseudo-molecule.

**Supplementary Table S4**: Overview of the whole-genome sequencing of *Brassica napus fancm* mutants and their wild type siblings

|  | *bnaC.fancm-1* | *bnaA.fancm-1* | *Bna.FANCM_1-1* plant #1 | *Bna.FANCM_1-1* plant #2 | *bna.fancm_1-1* plant #1 | *bna.fancm_1-1* plant #2 | Overall (non-redundant) |
| --- | --- | --- | --- | --- | --- | --- | --- |
| **# raw reads (x2)** | 39002790 | 68165044 | 152086280 | 160776792 | 129730122 | 145829854 |  |
| **# mapped reads (x2)^1^** | 25952676 (66%) | 45251400 (66%) | 96674067 (63%) | 101564924 (63%) | 82355829 (63%) | 92434914 (63%) |  |
| **# raw variant** | 788111 | 911223 | 1035755 | 1043957 | 1008628 | 1012422 | 1891159 |
| **# EMS mutation ^2^** | 3789 (0,48%) | 6373 (0,70%) | 5135 (0,50%) | 4373 (0,42%) | 6926 (0,70%) | 6504 (0,64%) | 20763 (1,09%) |
| **# EMS mutation in CDS** | 764 (20%) | 1414 (22%) | 1218 (24%) | 969 (22%) | 1638 (23%) | 1319 (20%) | 4714 (23%) |

^1^ : after rmdup/MM/Quality filtering

**^2^** : after quality/depth/SNP filtering

**Supplementary Table S5:** EMS-SNPS mutations targeting the coding sequence of genes experimentally shown to be involved in plant meiosis (other than FANCM)

| **Name** | **Bna_ID_(A_copy)** | **STOP** | **SPLICE** | **NON_SYN** | **Bna_ID_(C_copy)** | **STOP** | **SPLICE** | **NON_SYN** |
| --- | --- | --- | --- | --- | --- | --- | --- | --- |
| AGO9/AGO104 | BnaA02g05290D |  |  |  | NA |  |  |  |
| AGO9/AGO104 | BnaA10g14440D |  |  |  | BnaA10g14440D |  |  |  |
| SWI1/DYAD/AM1 | BnaA02g11280D |  |  |  | BnaC02g15840D |  |  |  |
| SWI1/DYAD/AM1 | BnaA10g08080D |  |  |  | BnaC09g27720D |  |  |  |
| MEI1/MCD1 | BnaA07g33480D |  |  |  | BnaC06g38030D |  |  |  |
| CDC45 | BnaA07g05080D |  |  |  | BnaCnng00060D |  |  |  |
| CDC45 | BnaA03g37670D |  |  |  | BnaC03g74180D |  |  |  |
| XRI1 | BnaA06g30010D |  |  |  | BnaC07g26700D |  |  |  |
| XRI1 | BnaA02g30390D |  |  |  | BnaCnng68010D |  |  |  |
| XRI1 | BnaA09g03120D |  |  |  | BnaC09g02540D |  |  |  |
| SPO11-1 | BnaA01g29970D |  |  |  | BnaC01g37870D |  |  |  |
| SPO11-2 | BnaA09g11340D |  |  |  | BnaC09g11780D |  |  |  |
| PRD1 | BnaA03g24590D |  |  |  | BnaC03g29300D |  |  |  |
| PRD2 | BnaA10g11740D |  |  |  | BnaC09g33470D |  |  |  |
| PRD2 | BnaA02g08030D |  |  |  | BnaC02g11140D |  |  |  |
| PAIR1/PRD3 | BnaA06g35310D |  |  |  | BnaC07g20440D |  |  |  |
| PAIR1/PRD3 | BnaA10g00300D |  |  |  | BnaC05g00390D |  |  |  |
| DFO | BnaA10g04520D |  |  |  | BnaC05g04880D |  |  |  |
| PHS1 | BnaAnng17480D |  |  |  | BnaC08g49930D |  |  |  |
| CRC1/PCH2 | BnaA01g14070D |  |  |  | BnaC01g16570D |  |  |  |
| **MRE11** | **BnaA10g07340D** | **0** | **0** | **1** | BnaC09g30690D |  |  |  |
| RAD50 | BnaA03g14800D |  |  |  | BnaC03g17840D |  |  |  |
| NBS1 | BnaA05g33420D |  |  |  | BnaCnng05860D |  |  |  |
| COM1/AtGR1 | BnaA04g05720D |  |  |  | BnaC04g28430D |  |  |  |
| RPA1A | BnaA07g04290D |  |  |  | BnaC07g47810D |  |  |  |
| RPA1C | BnaA02g23900D |  |  |  | chrC02:33823834 |  |  |  |
| RPA1C | BnaA09g17670D |  |  |  | BnaC09g18720D |  |  |  |
| RAD51 | BnaA10g14690D |  |  |  | BnaC09g37020D |  |  |  |
| RAD51 | BnaA03g08120D |  |  |  | BnaC03g10310D |  |  |  |
| RAD51B | BnaA04g16480D |  |  |  | BnaC04g39830D |  |  |  |
| RAD51C | BnaA05g04710D |  |  |  | BnaC04g04170D |  |  |  |
| RAD51D | BnaA08g29130D |  |  |  | BnaCnng55870D |  |  |  |
| XRCC3 | BnaA03g10270D |  |  |  | BnaC03g12950D |  |  |  |
| XRCC3 | NA |  |  |  | NA |  |  |  |
| BRCA2 (IV) | BnaA05g13130D |  |  |  | BnaC06g42060D |  |  |  |
| **BRCA2(V)** | **BnaA03g00230D** | **0** | **0** | **2** | BnaC03g00130D |  |  |  |
| **ATM** | **BnaA06g16810D** | **0** | **0** | **1** | BnaCnng09290D |  |  |  |
| **ATM** | **BnaA06g20800D** | **0** | **1** | **0** | **BnaC03g53270D** | **0** | **0** | **1** |
| **ATR** | **BnaA04g11880D** | **0** | **0** | **1** | **BnaC04g33820D** | **0** | **0** | **1** |
| MCM8 | BnaA05g28830D |  |  |  | BnaC05g43310D |  |  |  |
| DMC1 | BnaA01g24420D |  |  |  | BnaCnng14320D |  |  |  |
| DMC1 | BnaA03g36850D |  |  |  | NA |  |  |  |
| DMC1 | NA |  |  |  | BnaC05g29630D |  |  |  |
| AHP2/HOP2 | BnaA06g07430D |  |  |  | BnaC05g08850D |  |  |  |
| MND1 | BnaA03g49390D |  |  |  | BnaC07g41780D |  |  |  |
| SDS | BnaA06g09550D |  |  |  | BnaC05g10920D |  |  |  |
| **SDS** | BnaA09g45480D |  |  |  | **BnaC08g39330D** | **0** | **0** | **1** |
| SDS | BnaA08g31410D |  |  |  | BnaC08g16420D |  |  |  |
| FIGL1 | BnaA02g28640D |  |  |  | BnaC02g36660D |  |  |  |
| MSH4 | BnaA08g08260D |  |  |  | BnaCnng35120D |  |  |  |
| MSH5 | BnaA05g20450D |  |  |  | BnaCnng46130D |  |  |  |
| ZIP4 | BnaA06g30240D |  |  |  | BnaC07g26460D |  |  |  |
| MER3/RCK | BnaA02g29040D |  |  |  | BnaC02g37070D |  |  |  |
| HEI10 | BnaA06g00910D |  |  |  | BnaC06g06360D |  |  |  |
| HEI10 | BnaA05g14420D |  |  |  | BnaCnng35870D |  |  |  |
| HEI10 | NA |  |  |  | BnaC03g69660D |  |  |  |
| SHOC1 | BnaA03g12670D |  |  |  | BnaC03g15550D |  |  |  |
| PTD | BnaA06g38110D |  |  |  | BnaC05g49280D |  |  |  |
| MLH1 | BnaA03g23940D |  |  |  | BnaC03g28480D |  |  |  |
| **MLH3** | **BnaA03g53380D** | **0** | **1** | **0** | BnaC07g45660D |  |  |  |
| RBR1 | BnaA05g26760D |  |  |  | BnaC05g40780D |  |  |  |
| RBR1 | BnaA01g30730D |  |  |  | BnaCnng22930D |  |  |  |
| **RBR1** | **BnaA03g32200D** | **0** | **0** | **1** | BnaC03g37490D |  |  |  |
| MUS81 | BnaA09g20790D |  |  |  | NA |  |  |  |
| RMI1 | BnaA09g06650D |  |  |  | BnaC09g05950D |  |  |  |
| Topo3alpha | BnaA02g33960D |  |  |  | BnaC02g42810D |  |  |  |
| Topo3alpha | BnaA06g23060D |  |  |  | BnaC03g50350D |  |  |  |
| MHF1 | BnaA03g13400D |  |  |  | BnaC03g16220D |  |  |  |
| MHF2 | BnaA07g34380D |  |  |  | BnaC06g39210D |  |  |  |
| MHF2 | NA |  |  |  | BnaC03g70530D |  |  |  |
| RECQ4A | BnaA09g47850D |  |  |  | BnaC08g42280D |  |  |  |
| RECQ4A | BnaA08g25530D |  |  |  | BnaC08g14630D |  |  |  |
| RECQ4B | BnaA09g14010D |  |  |  | BnaC09g14340D |  |  |  |
| RFC1 | BnaA02g05420D |  |  |  | BnaC08g49560D |  |  |  |
| **XRCC2** | **BnaA09g06860D** | **0** | **0** | **1** | BnaC09g06360D |  |  |  |
| MSH2 | BnaA03g34810D |  |  |  | BnaCnng23780D |  |  |  |
| CDKG1 | BnaCnng61700D |  |  |  | BnaC02g42490D |  |  |  |
| ARP6 | BnaA08g00100D |  |  |  | BnaC03g71020D |  |  |  |
| **AXR1** | **BnaA10g03160D** | **0** | **0** | **1** | BnaC05g03140D |  |  |  |
| AXR1 | BnaA08g28190D |  |  |  | BnaC08g01230D |  |  |  |
| AXR1 | NA |  |  |  | BnaC05g03160D |  |  |  |
| PSS1 | NA |  |  |  | NA |  |  |  |
| ASY1 | BnaA07g26350D |  |  |  | BnaC06g28450D |  |  |  |
| ASY1 | NA |  |  |  | BnaC06g26770D |  |  |  |
| PAIR3/ASY3 | BnaA05g00870D |  |  |  | BnaC04g00500D |  |  |  |
| PAIR3/ASY3 | BnaA03g21580D |  |  |  | BnaC03g72410D |  |  |  |
| ZYP1/ZEP1 | BnaA07g34050D |  |  |  | BnaC06g38690D |  |  |  |
| ZYP1/ZEP1 | BnaA07g20670D |  |  |  | BnaC06g20530D |  |  |  |
| ASY4 | BnaA04g19810D |  |  |  | BnaC04g44200D |  |  |  |
| ASY4 | BnaA05g09850D |  |  |  | BnaCnng05260D |  |  |  |
| SYN1/DIF1/REC8 | BnaA10g24970D |  |  |  | BnaC09g49900D |  |  |  |
| **SCC2** | BnaA10g18670D |  |  |  | **BnaC09g42410D** | **0** | **0** | **1** |
| SCC2 | BnaA02g02660D |  |  |  | BnaC02g06100D |  |  |  |
| SCC3 | BnaA05g00110D |  |  |  | BnaC04g51720D |  |  |  |
| SGO1 | BnaA01g31730D |  |  |  | NA |  |  |  |
| **AtSGO1 (a duplic)** | **BnaA10g25920D** | **0** | **0** | **1** | BnaCnng10160D |  |  |  |
| AtSGO1 (a duplic) | BnaA03g01210D |  |  |  | BnaC03g71160D |  |  |  |
| SYN2/RAD21.1 | BnaA04g10640D |  |  |  | BnaC04g32940D |  |  |  |
| SYN2/RAD21.1 | NA |  |  |  | BnaC06g13250D |  |  |  |
| SYN3/RAD21.2 | BnaA09g37900D |  |  |  | BnaC08g29760D |  |  |  |
| SYN3/RAD21.2 | NA |  |  |  | BnaC04g23000D |  |  |  |
| SYN4/RAD21.3 | BnaA10g18050D |  |  |  | BnaC09g41550D |  |  |  |
| SYN4/RAD21.3 | BnaA02g03030D |  |  |  | BnaC02g06490D | 0 | 0 | 1 |
| SYN4/RAD21.3 | BnaA03g05900D |  |  |  | BnaC03g07660D |  |  |  |
| SMC1/TITAN8 | BnaA09g34490D |  |  |  | BnaC08g25430D |  |  |  |
| SMC1/TITAN8 | BnaA03g37440D |  |  |  | BnaC03g43960D |  |  |  |
| SMC1/TITAN8 | NA |  |  |  | BnaC04g26130D |  |  |  |
| AtSMC3 | NA |  |  |  | BnaC04g38670D |  |  |  |
| AtSMC3 | BnaA03g22510D |  |  |  | BnaC03g72770D |  |  |  |
| AESP | BnaA01g12740D |  |  |  | BnaC01g14470D |  |  |  |
| AESP | BnaA03g45660D |  |  |  | BnaC07g37760D |  |  |  |
| PAN1 | BnaA05g25290D |  |  |  | NA |  |  |  |
| PAN1 | NA |  |  |  | BnaC01g37170D |  |  |  |
| PAN1 | BnaA03g33260D |  |  |  | BnaC03g38350D |  |  |  |
| PAN2 | BnaA10g20340D |  |  |  | BnaC09g44370D |  |  |  |
| CTF7/ECO1 | BnaA03g51450D |  |  |  | BnaC07g50930D |  |  |  |
| CTF7/ECO1 | BnaA08g12500D |  |  |  | BnaC03g67630D |  |  |  |
| CDKA;1 | NA |  |  |  | BnaC08g20470D |  |  |  |
| CDKA;1 | BnaA01g20470D |  |  |  | BnaC01g25750D |  |  |  |
| CDKA;1 | BnaA06g18950D |  |  |  | BnaC03g75260D |  |  |  |
| CDKA;1 | NA |  |  |  | BnaC03g52880D |  |  |  |
| TAM/CYCA1;2 | BnaA07g33530D |  |  |  | BnaC06g38120D |  |  |  |
| TAM/CYCA1;2 | BnaA05g23170D |  |  |  | NA |  |  |  |
| TAM/CYCA1;2 | NA |  |  |  | BnaC02g13720D |  |  |  |
| MS5/TDM | BnaA03g44650D |  |  |  | BnaC07g36490D |  |  |  |
| OSD1 | BnaA09g37090D |  |  |  | BnaC08g28850D |  |  |  |
| UVI4 | BnaA05g02710D |  |  |  | BnaC04g02550D |  |  |  |
| SMG7 | BnaA10g15730D |  |  |  | BnaC09g38310D |  |  |  |
| SMG7 | BnaA02g04210D |  |  |  | BnaC02g44260D |  |  |  |
| JASON | BnaA10g04350D |  |  |  | BnaC05g04570D |  |  |  |
| **JASON** | **BnaA08g28700D** | **0** | **0** | **1** | BnaC08g01870D |  |  |  |
| PS1 | NA |  |  |  | BnaC06g08200D |  |  |  |
| PS1 | BnaA08g06420D |  |  |  | BnaC08g06940D |  |  |  |
| PS1 | BnaA10g09600D |  |  |  | BnaC09g31940D |  |  |  |
| ATK1/KATA/KIN14a | NA |  |  |  | NA |  |  |  |
| MMD/DUET | BnaA02g12370D |  |  |  | BnaCnng19010D |  |  |  |
| MPK4 | BnaA03g26950D |  |  |  | BnaC03g31910D |  |  |  |
| TES/NACK2/STD | BnaA06g19570D |  |  |  | BnaCnng11920D |  |  |  |
| **ASK1** | **BnaA07g32230D** | **0** | **0** | **1** | BnaC06g36600D |  |  |  |
| ASK1 | BnaA02g17620D |  |  |  | BnaC02g23320D |  |  |  |
| ASK1 | BnaA07g21520D |  |  |  | BnaC06g22030D |  |  |  |
| PCH2 | NA |  |  |  | BnaC01g16570D |  |  |  |

These mutations are predicted to affect protein function according to the SIFT algorithm

# Supplementary Figures

| **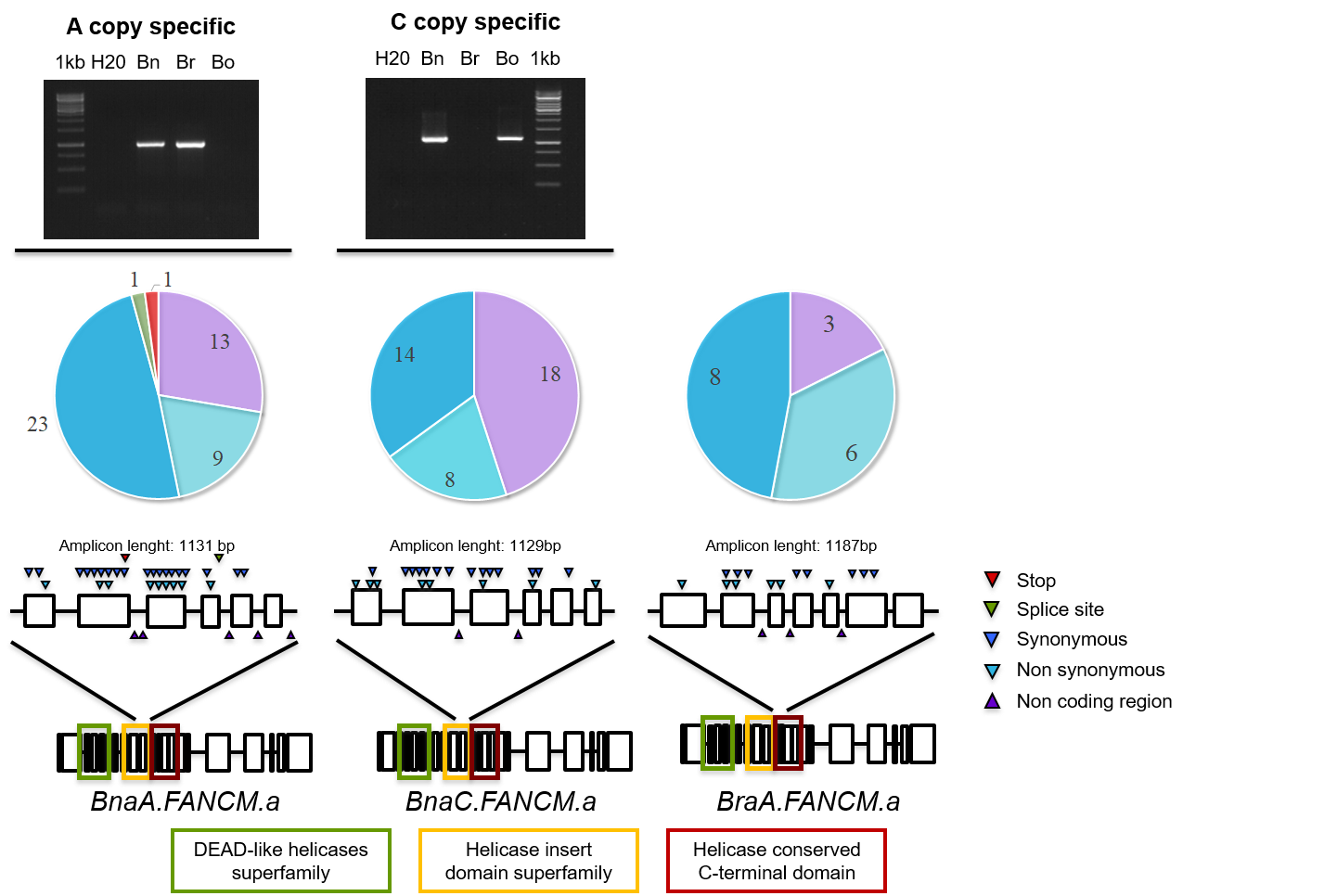**  **Supplementary Figure S1. Overview of the *fancm* mutations identified by TILLING in *Brassica napus* and *B. rapa***  Amplification of *BnaA.FANCM* and *BnaC.FANCM* was performed using pairs of copy specific primers that amplify either the A copy of FANCM both in *Brassica napus* (Bn) and *B. rapa* (Br) or the C copy of FANCM both in *B. napus* and *B. oleracea* (Bo). For each homolog of *FANCM*, the intron-exon composition of the gene models is represented along with the approximate position of the helicase domains (lower diagram). The mutations identified are represented by arrowheads along a zoom of the screened region, i.e., the bipartite helicase domain of FANCM (around 1kb long; upper diagram). Distribution of mutations with regards to their type is given by the pie charts for each gene model. |
| --- |


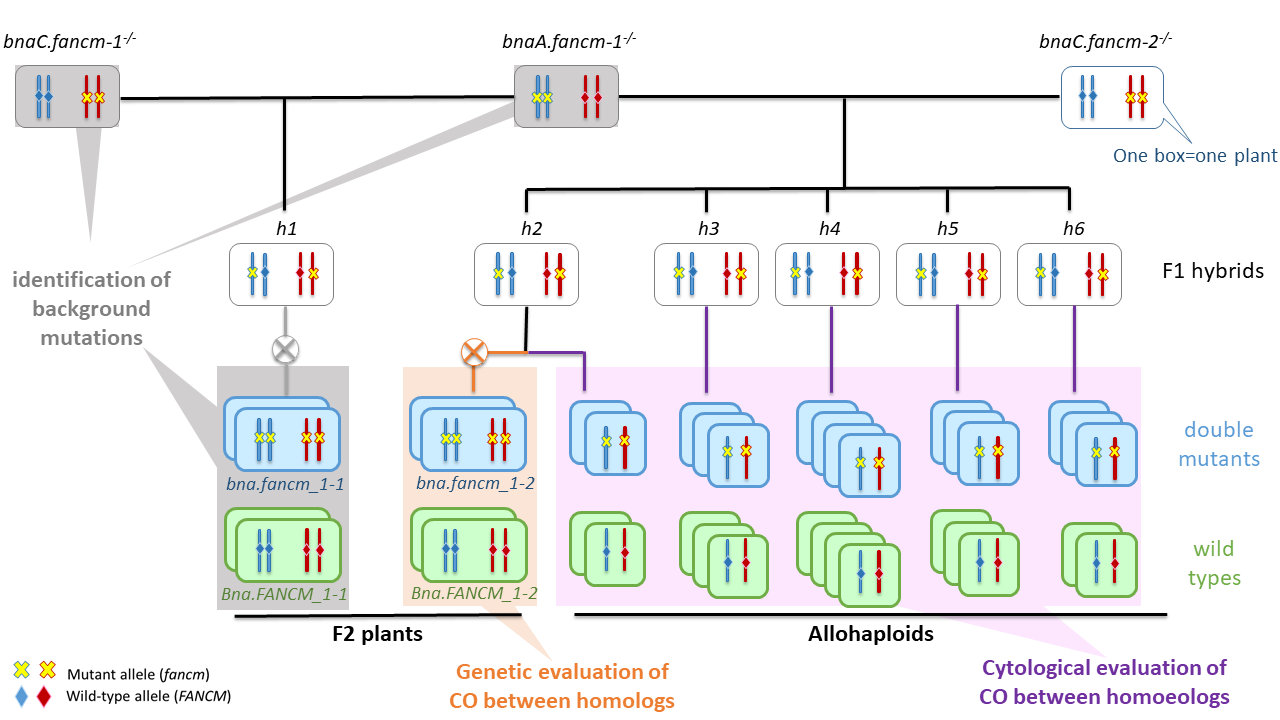


**Supplementary Figure 2. Genealogy of the plants used to assess crossover (CO) frequencies**

For each plant (each represented by a box), the allelic version of *BnaA.FANCM* and *BnaC.FANCM* is represented (WT: diamond; mutant=cross). F1 hybrids (h1 and h2) were selfed to produce F2 progenies among which double homozygous mutants for *FANCM* (*bna.fancm1-1* and *bna.fancm1-2)* (blue) and their WT siblings *(Bna.FANCM 1-1* and *Bna.FANCM 1-2*) (green) were selected. These plants were selfed to produce F3 progenies, from which crossover (CO) formation between homologous chromosomes was assessed genetically. Our initial aim was to replicate the experiment using two different combinations of mutant and WT alleles (*bna.fancm1-1* vs *Bna.FANCM 1-1* and *bna.fancm1-2^-^* vs *Bna.FANCM 1-2*) and, for each replication, two different double mutants and WT siblings. Unfortunately, the first combination of mutations (*bna.fancm1-1*) proved not to alter FANCM function. These plants, which were obtained first, were used to identify the EMS-SNP segregating from *bnaA.fancm 1^-/-^,* and then discarded. F1 hybrids (h2 to h6) were also used for microspore culture (purple line) to generate allohaploids segregating for the WT and mutant alleles of *BnaA.FANCM* and *BnaC.FANCM.* A minimum of two double mutants and two WT siblings from the same progeny were retained to assess and compare crossover (CO) formation between homoeologous chromosomes in the two genetic backgrounds.


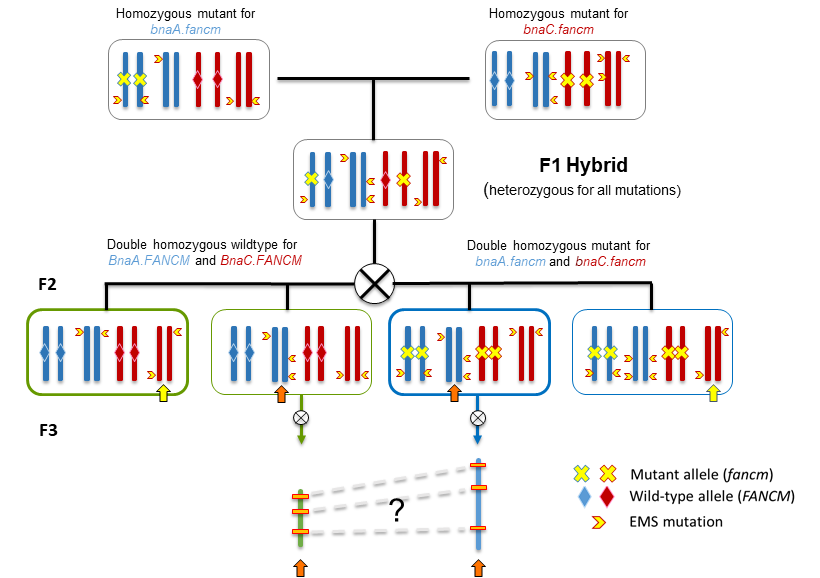


**Supplementary Figure 3. Experimental design to assess homologous recombination frequencies in *Brassica napus***

Here are represented plants (each represented by a box) that are defective for either *BnaA.FANCM* or *BnaC.FANCM*, the F1 hybrid produced by crossing these two plants and four F2 offsprings that are either defective for the two copies of FANCM (with a blue border) or WT (with a green border). For each plant, four pairs of homologous chromosomes are shown; they include A05 and C05, where *BnaA.FANCM* and *BnaC.FANCM* are located, and two random additional pairs of homologues from the A (blue) and the C (red) genomes (the sister chromatids are not represented). Background EMS mutations (yellow chevrons) segregate in the selfed progeny of the F1 hybrid and define intervals in which recombination frequencies can be measured and compared in the F3 progenies (genetic map and coloured arrows).


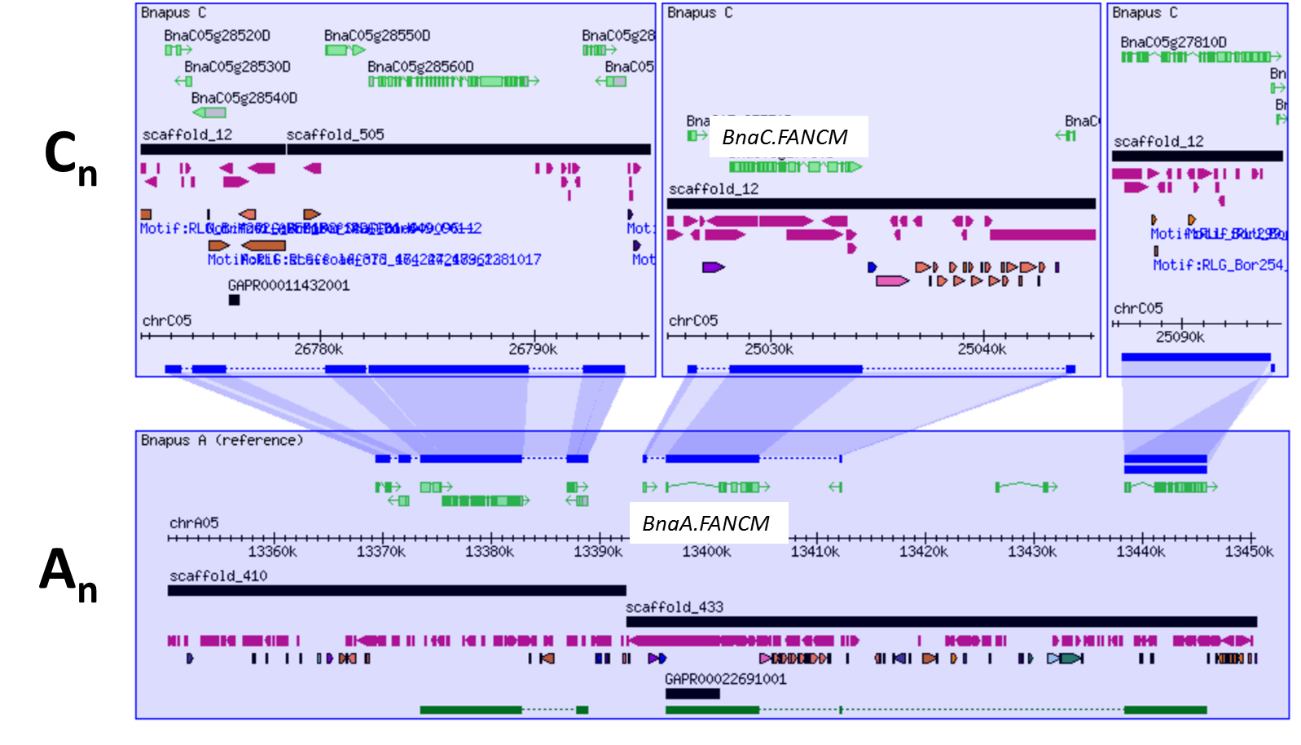


**Supplementary Figure 4. *BnaA.FANCM* and *BnaC.FANCM* are located in syntenic regions.**

Here are provided outputs from syntenic comparisons between homoeologous A (bottom) and C (top) regions surrounding BnaA.FANCM and BnaC.FANCM. This figure was generated using the synteny tool within the Genoscope Brassica napus genome browser (http://www.genoscope.cns.fr/brassicanapus/cgi-bin/gbrowse_syn/colza/). Gene models are represented by colored arrows. Pairs of homoeologues are represented by thick blue lines connected by shaded boxes.


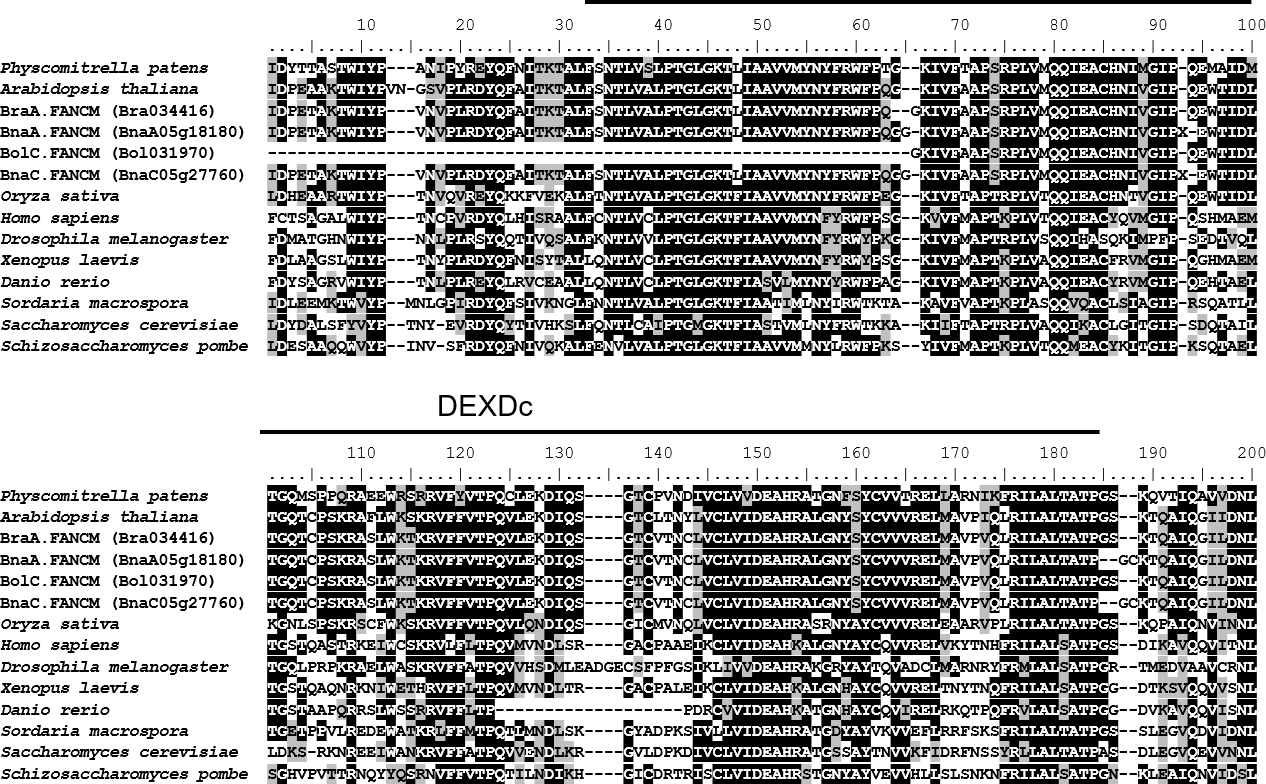

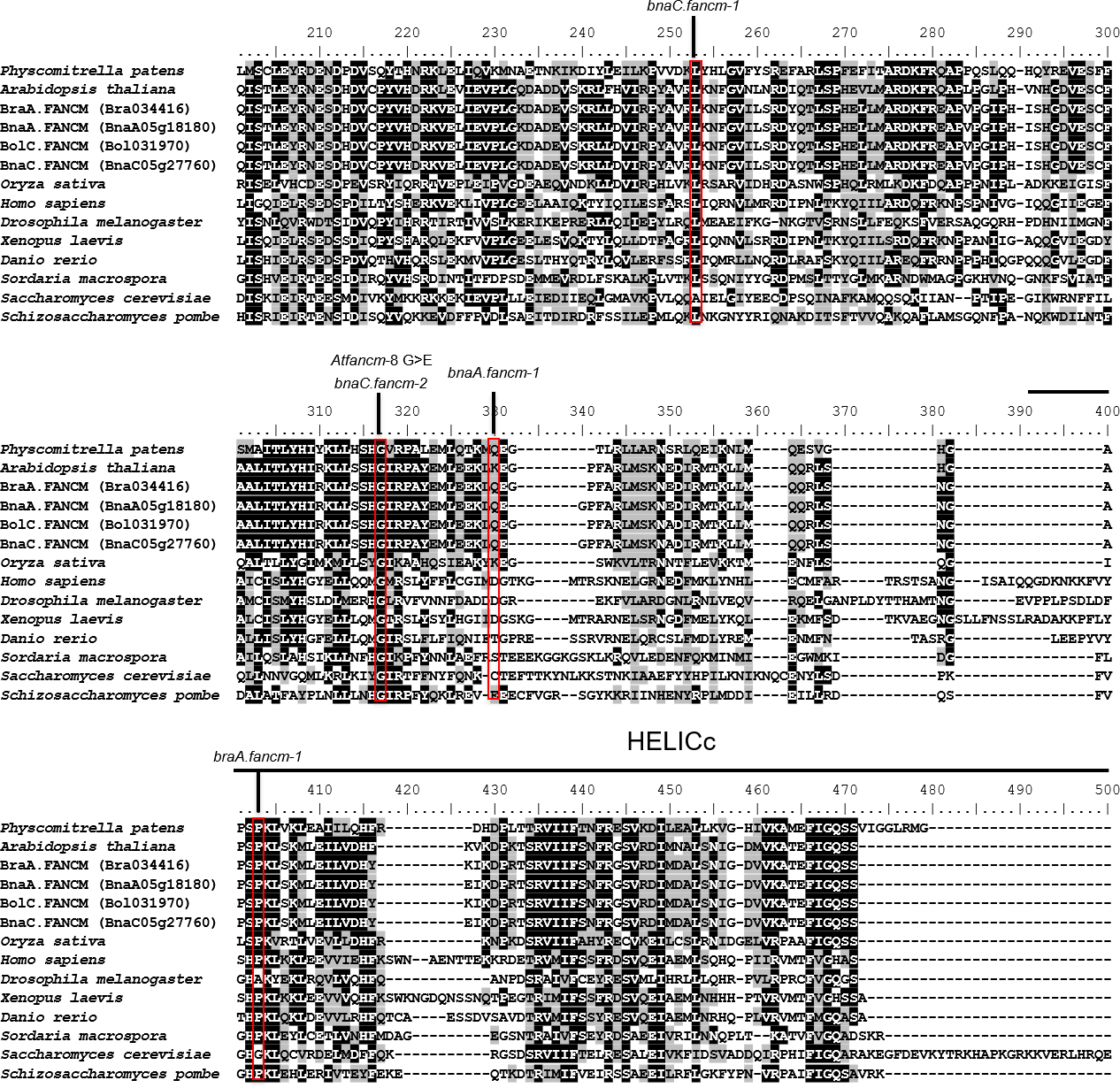


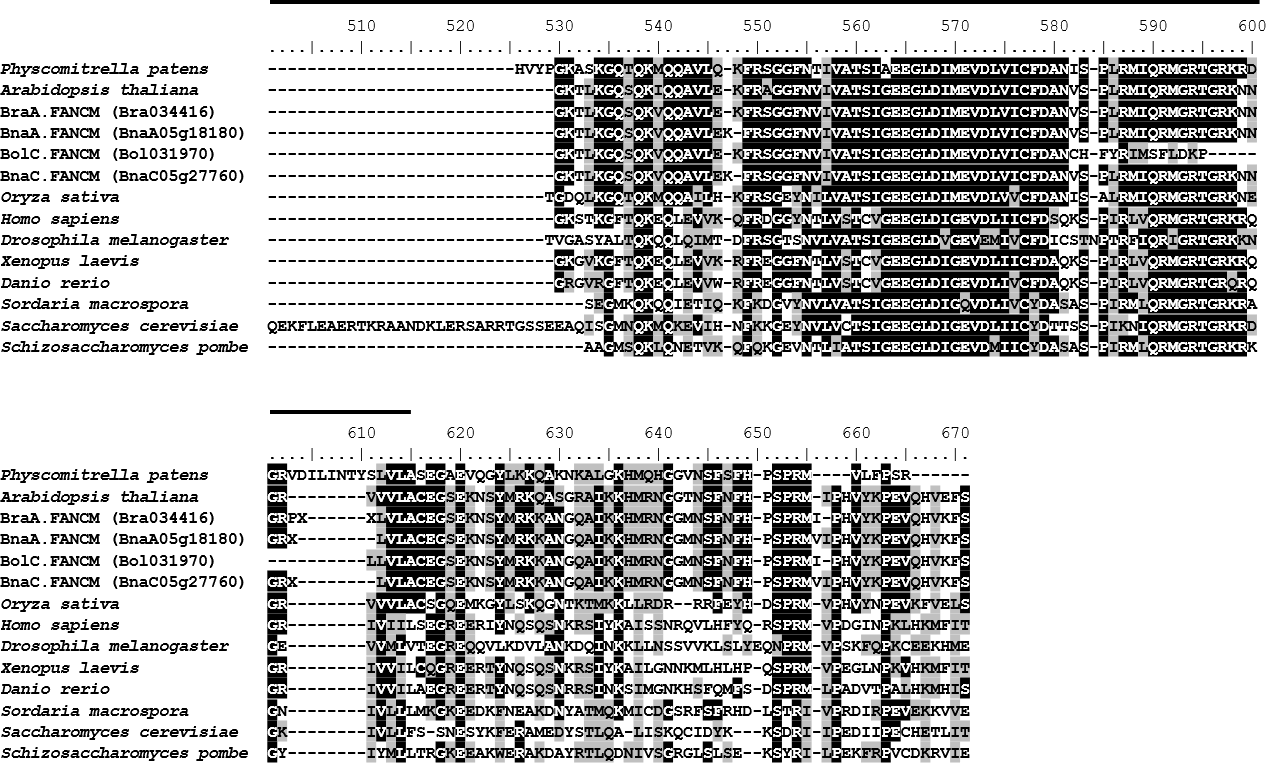


**Supplementary Figure 5. ClustalW multiple alignment of the helicase region of FANCM in *Arabidopsis thaliana*, *Brassica* and some representative eukaryotes**

The positions of the mutations selected in this study are shown along the N-terminal bipartite SF2 helicase domain of FANCM. *Physcomitrella patens* XP_001753469.1; *Arabidopsis thaliana* NM_001198212; *Oryza sativa* AAX96303.1; *Homo sapiens* FANCM NP_065988.1; *Drosophila melanogaster* NP_650971.2; *Xenopus laevis* NP_001171151.1; *Danio rerio* NP_001107132.1; *Sordaria macrospora* XP_003348274.1; *Saccharomyces cerevisiae* Mph1 NP_012267.1; *Schizosaccharomyces pombe* Fml1 Q9UT23.2.


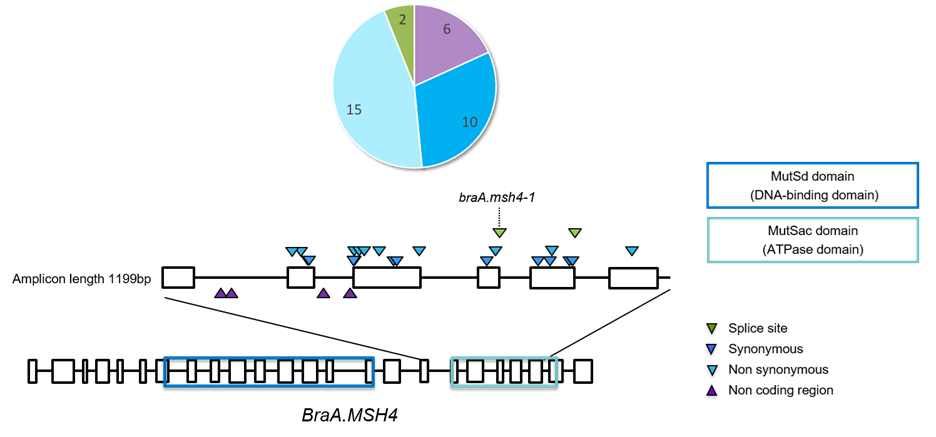


**Supplementary Figure 6. Overview of the *msh4* mutations identified by TILLING in *Brassica rapa***

A black arrow points to the position of the mutation *braA.msh4-1* on *BraA.MSH4*. *braA.msh4-1* induces a substitution in the acceptor site of the 19^th^ exon; the composition intron (line) exon (box) of *BraA.MSH4* is given. *BrA.MSH4* coding sequence contains 792 aminoacids.


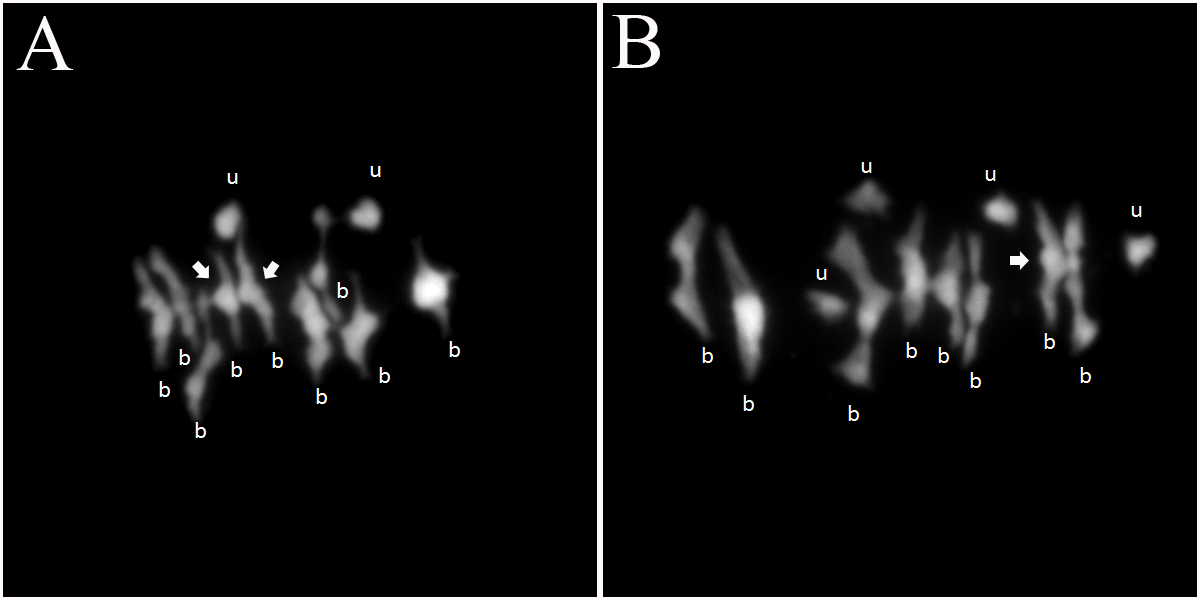


**Supplementary Figure 7. Representative Metaphase I plates in the double mutant *braA.msh4-1^-/-^ braA.fancm-1^-/-^***

Metaphase I plates in the double mutant *braA.msh4-1^-/-^ braA.fancm-1^-/-^*  show (A) one meiocyte with 9 bivalents (b) plus one pair of univalent (u) and (B) one meiocyte with 8 bivalents plus two pairs of univalent. Note that some bivalents (pointed by arrows) show a shape that is suggestive of the occurrence of multiple crossovers along chromosome arms, as they appear to be more compacted than in wild types. This shape is reminiscent of what Fernandes et al. (2017) observed in *A. thaliana* hyper-recombinant mutants. Scale bar = 10um


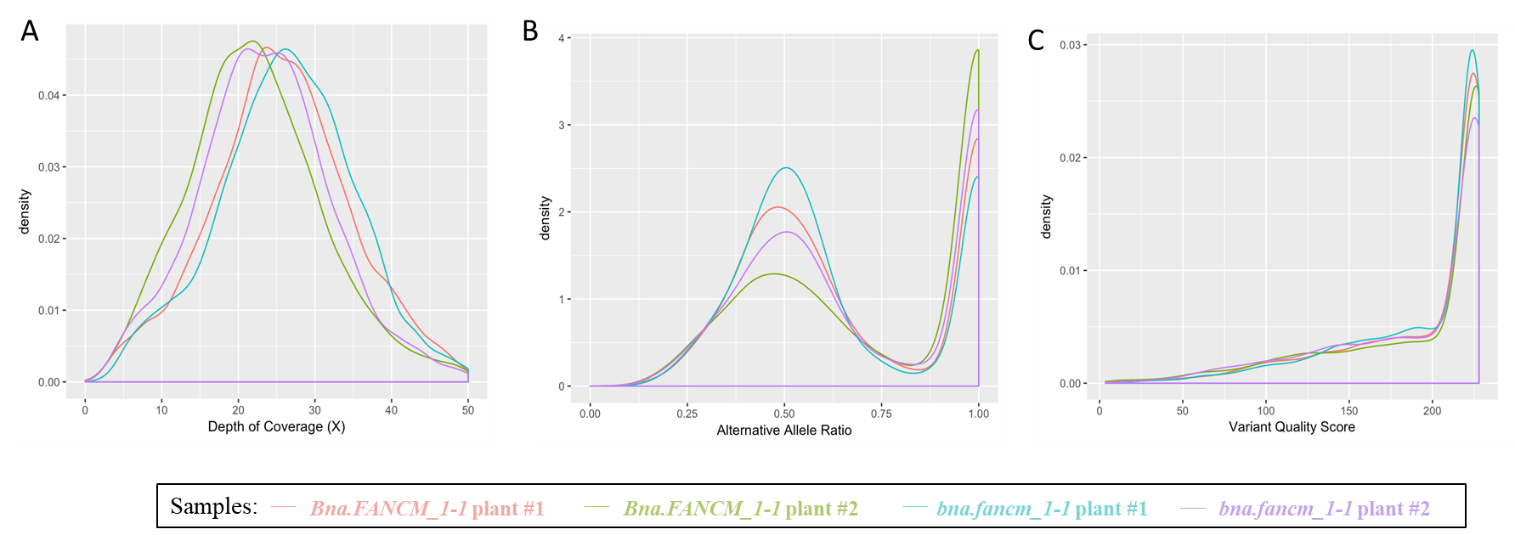


**Supplementary Figure 8. Quality control of detected EMS mutations in CDS regions**

(A): Distribution of read depth at mutated positions, (B): Distribution of alternative allele ratio (0.5 means heterozygote; 1 means homozygote for a mutation) and (C) Distribution of quality score assigned to mutations (as defined by Li (2011) Bioinformatics. 27(21): 2987–2993).

.

**Supplementary Figure 9. Chromosomal distribution of EMS mutations affecting pairs of homoeologous genes and detected in the homozygous state at both loci in at least one plant.**

Arrows indicate the position of mutations along the 19 *B. napus* chromosomes (A01-A10 and C01-C09). Mutations inherited from *bnaC.fancm-1* (above) and *bnaA.fancm-1* (below) are represented on either side of chromosomes. Arrows of the same color represent mutations targeting pairs of homoeologues (the gene ID are provided on the right).
